# Overlapping open reading frames strongly reduce human and yeast *STN1* gene expression and affect telomere function

**DOI:** 10.1371/journal.pgen.1007523

**Published:** 2018-08-01

**Authors:** Victoria Torrance, David Lydall

**Affiliations:** Institute for Cell and Molecular Biosciences, Newcastle University Medical School, Newcastle upon Tyne, Tyne and Wear, United Kingdom; Stanford University School of Medicine, UNITED STATES

## Abstract

The levels of telomeric proteins, such as telomerase, can have profound effects on telomere function, cell division and human disease. Here we demonstrate how levels of Stn1, a component of the conserved telomere capping CST (Cdc13, Stn1, Ten1) complex, are tightly regulated by an upstream overlapping open reading frame (oORF). In budding yeast inactivation of the *STN1* oORF leads to a 10-fold increase in Stn1 levels, reduced telomere length, suppression of *cdc13-1* and enhancement of *yku70Δ* growth defects. The *STN1* oORF impedes translation of the main ORF and reduces *STN1* mRNA via the nonsense mediated mRNA decay (NMD) pathway. Interestingly, the homologs of the translation re-initiation factors, MCT-1^Tma20^/DENR^Tma22^ also reduce Stn1 levels via the oORF. Human *STN1* also contains oORFs, which reduce expression, demonstrating that oORFs are a conserved mechanism for reducing Stn1 levels. Bioinformatic analyses of the yeast and human transcriptomes show that oORFs are more underrepresented than upstream ORFs (uORFs) and associated with lower protein abundance. We propose that oORFs are an important mechanism to control expression of a subset of the proteome.

## Author summary

Telomeres are special structures at the ends of linear chromosomes that help protect the genetic information that chromosomes carry. The levels of telomere proteins are important and can affect diseases such as cancer and ageing. The CST complex is comprised of three proteins and binds human and yeast telomeres. Levels of Stn1, a very low abundance protein, are of particular importance to telomere function in yeast cells. There are many ways to affect protein levels but little was understood about how Stn1 levels are controlled. We show that levels of Stn1 in yeast and human cells are reduced by the presence of an upstream overlapping open reading frame (oORF). Cells lacking the oORF have short telomeres and increased fitness when combined with a defect in the Stn1-partner protein, Cdc13. Interestingly, in another telomere defective context, *yku70*Δ cells missing the *STN1*-oORF are less fit. We show that the oORF reduces Stn1 levels by stimulating nonsense mediated mRNA decay and by reducing translation. More generally, genome-wide computational analysis shows that oORFs were strongly selected against during evolution and when present are associated with low protein abundance. We propose that oORFs are a powerful mechanism to regulate protein expression and function.

## Introduction

The bulk of the genome is duplicated precisely once each cell cycle but telomeres are replicated differently. DNA polymerases cannot replicate the ends of linear molecules and therefore different strategies are needed to replicate telomeric DNA. The majority of eukaryotes use a reverse transcriptase based enzyme, telomerase, to elongate DNA at telomeric ends. Telomerase activity is tightly regulated. Most human somatic cells express low levels of telomerase, therefore the telomeres shorten with each cell cycle, eventually leading to cell cycle arrest and senescence [1]. In contrast, many cancers over-express telomerase and hyper-elongate telomeres, a process that facilitates uncontrolled cell division. Indeed, point mutations in the telomerase (*TERT*) promoter, which increase TERT expression, are the most commonly identified non-coding mutations found in human cancer [2, 3].

Yeast cells also express telomerase and maintain stable telomere length. Typically, yeast telomeric DNA shortens over several cell cycles, due to the end replication problem, or sometimes more acutely, due to DNA replication failure. Short telomeres are preferential substrates for telomerase ensuring that in yeast telomere length is comparatively stable [4, 5]. In addition to telomerase, numerous other proteins contribute to telomere length homeostasis. The conserved CST complex has affinity for the G-rich single stranded DNA (ssDNA) at the very 3’ terminus of telomeres [6]. CST is encoded by *CTC1*, *STN1* and *TEN1* in human cells and mutations in *CTC1* and *STN1* are associated with Coats plus disease, one of the heritable telomere syndromes [7, 8]. In budding yeast, the equivalent proteins are Cdc13, Stn1 and Ten1, and each is essential for telomere function and cell viability. CST has many functions, including protecting telomeres from the harmful effects of the DNA damage response, regulating telomerase activity and recruiting DNA Pol α to complete lagging strand replication [9, 10].

Individual CST proteins play complex roles in telomerase recruitment, telomerase inhibition and telomere capping [9, 11]. In particular, there is evidence that levels of Stn1 are important. Increased expression of *STN1* leads to short telomeres, which is thought to be due to inhibition of telomerase [12, 13]. Additionally, overexpression of Stn1 can compensate for partial loss of telomere capping in strains defective in Cdc13 [14]. Finally, there is evidence that increased levels of Stn1 inactivate the S phase checkpoint that responds to genome-wide stalled replication forks [15]. Thus, Stn1 levels affect telomere function and the DNA damage response.

Protein levels can be affected by numerous mechanisms affecting transcription, translation or degradation and each type of mechanism has associated costs and benefits [16]. Interestingly, *STN1* transcript levels are strongly reduced by the nonsense mediated mRNA decay pathway (NMD) and *STN1* is among the top 2% of NMD targets [12, 17]. This indicates that RNA degradation is an important mechanism for reducing Stn1 levels. However, how the NMD pathway targets *STN1* transcripts is unclear. Early studies indicated that the *STN1* upstream regulatory sequence (URS, 300 bases upstream of the *STN1* CDS) conferred NMD-dependent control [12]. More recently it was suggested that NMD targets *STN1* via a programmed -1 ribosomal frameshifting signal in the C-terminal half of the CDS [18].

Here we establish that the yeast *STN1* transcript is targeted by NMD principally because it contains an upstream overlapping ORF (oORF), a special class of upstream ORF (uORF). Furthermore, this oORF is also the route by which the conserved translation re-initiation heterodimer, Tma20^MCT-1^/Tma22^DENR^ reduces expression of *STN1*. Importantly, inactivation of the *STN1* oORF has numerous strong effects on yeast telomere function. Interestingly, the human *STN1* transcript is also dramatically reduced by the presence of an oORF, suggesting that there is conservation of oORF-dependent mechanisms for reducing Stn1 levels. uORF translation is widespread and associated with reduced expression in both yeast and mammalian cells [19, 20]. Our analyses of yeast and human genomes shows that oORFs have been more heavily selected against than uORFs, and are associated with lower protein abundance. *STN1* is therefore likely to be just one striking example of how an oORF affects levels of a key regulatory protein.

## Results

### The *STN1* overlapping open reading frame reduces expression

**Fig 1:**
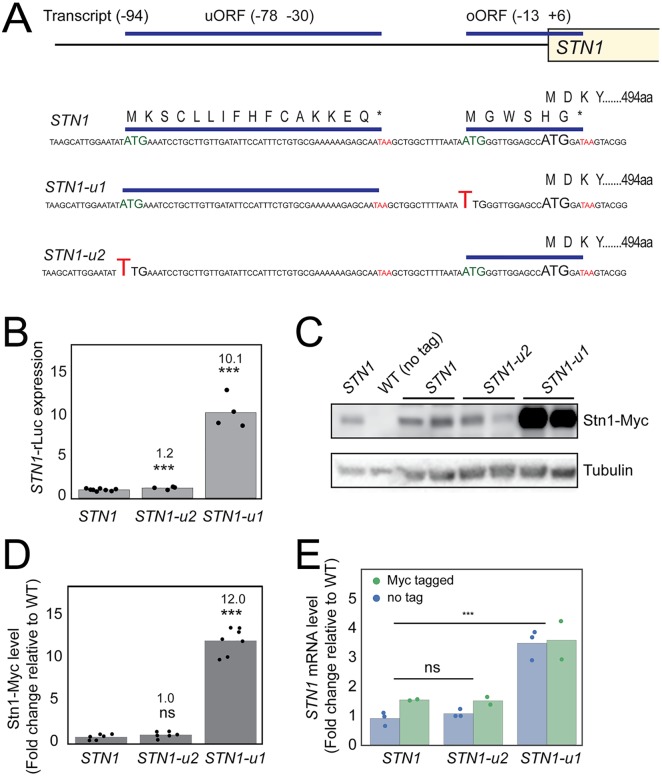
The *STN1* overlapping open reading frame reduces expression. A) Cartoon of the *STN1* upstream regulatory sequence (URS). *STN1-u1* and *STN1-u2* are point mutations in the initiation codons of the oORF and uORF, respectively. B) A dual luciferase reporter plasmid was used to measure the effects of *STN1* URSs on gene expression. The ratio of Rluc activity (*STN1*) over Fluc activity (*PGK1*) was calculated to obtain the normalized Rluc activity. *STN1* Rluc activity was given a value of 1 and *STN1-u1* and *STN1-u2* were calculated relative to this. Each point represents a single measurement from an independent transformant clone. C) Western blot analysis of Stn1-Myc and tubulin levels. D) Quantification of western blots, as shown in C, and S9 Fig. Stn1-Myc levels were normalized to the loading control, tubulin. The WT strain was given a value of 1 and other values were calculated relative to this. E) *STN1* transcript levels were measured by RT-qPCR and normalized to a loading control, *BUD6*. The WT strain was given a value of 1 and other values were calculated relative to this. Each point on the plot represents an independent RNA preparation (the mean of triplicate measurements). *P* values were calculated using an unpaired t-test (***) *P* < 0.001.

It is well established that Stn1 levels are affected by NMD and affect telomere function [12]. We noticed that the *STN1* upstream regulatory sequence (URS) has two uORFs. These encode a 16 amino acid uORF and a 6 amino acid oORF, the latter terminating 2 bases after the *STN1* coding sequence (CDS) start codon (Fig 1A). To examine the effects of the uORF and oORF on expression, the initiation codons were mutated, creating *STN1-u2* and *STN1-u1*, respectively. *STN1-u2*, 78 nucleotides upstream of the *STN1* CDS, is a point mutation in the uORF initiation codon while *STN1-u1* (13 nucleotides upstream) is a point mutation in the oORF initiation codon (Fig 1A). A dual luciferase reporter plasmid was used to measure *STN1* URS driven expression. *STN1-u2* slightly increased expression (1.2 fold) while *STN1-u1* dramatically increased expression (10 fold) (Fig 1B). These data suggest that the *STN1* oORF is a strong inhibitor of Stn1 expression, while the *STN1* uORF has a milder effect.

We next determined the effects of the uORF and oORF in the genome. *STN1-u1* and *STN1-u2* point mutations were introduced into the *STN1* chromosomal locus, upstream of a C-terminal epitope-tagged *STN1* construct. Western blot analysis of Myc-tagged Stn1 protein, which retains function and has been widely used [21, 22], confirmed that *STN1-u1* has a much stronger effect on gene expression (12-fold) than *STN1-u2* (no detectable increase) (Fig 1C and 1D). These results, concordant with the luciferase assays, allow us to conclude that the *STN1* oORF is a potent inhibitor of gene expression.

The *STN1* oORF could reduce expression by impeding translation, reducing mRNA levels, or both. To test these possible mechanisms *STN1* transcript levels were measured. A 4-fold increase in *STN1* transcript levels was caused by *STN1-u1* (Fig 1E), significantly less than its 12-fold effect on protein levels (Fig 1D). This difference suggests that the *STN1* oORF affects gene expression by reducing transcript levels and translation of these transcripts.

### *STN1-u1* improves fitness of *cdc13-1* cells, decreases fitness of *yku70Δ* cells and causes short telomeres

**Fig 2:**
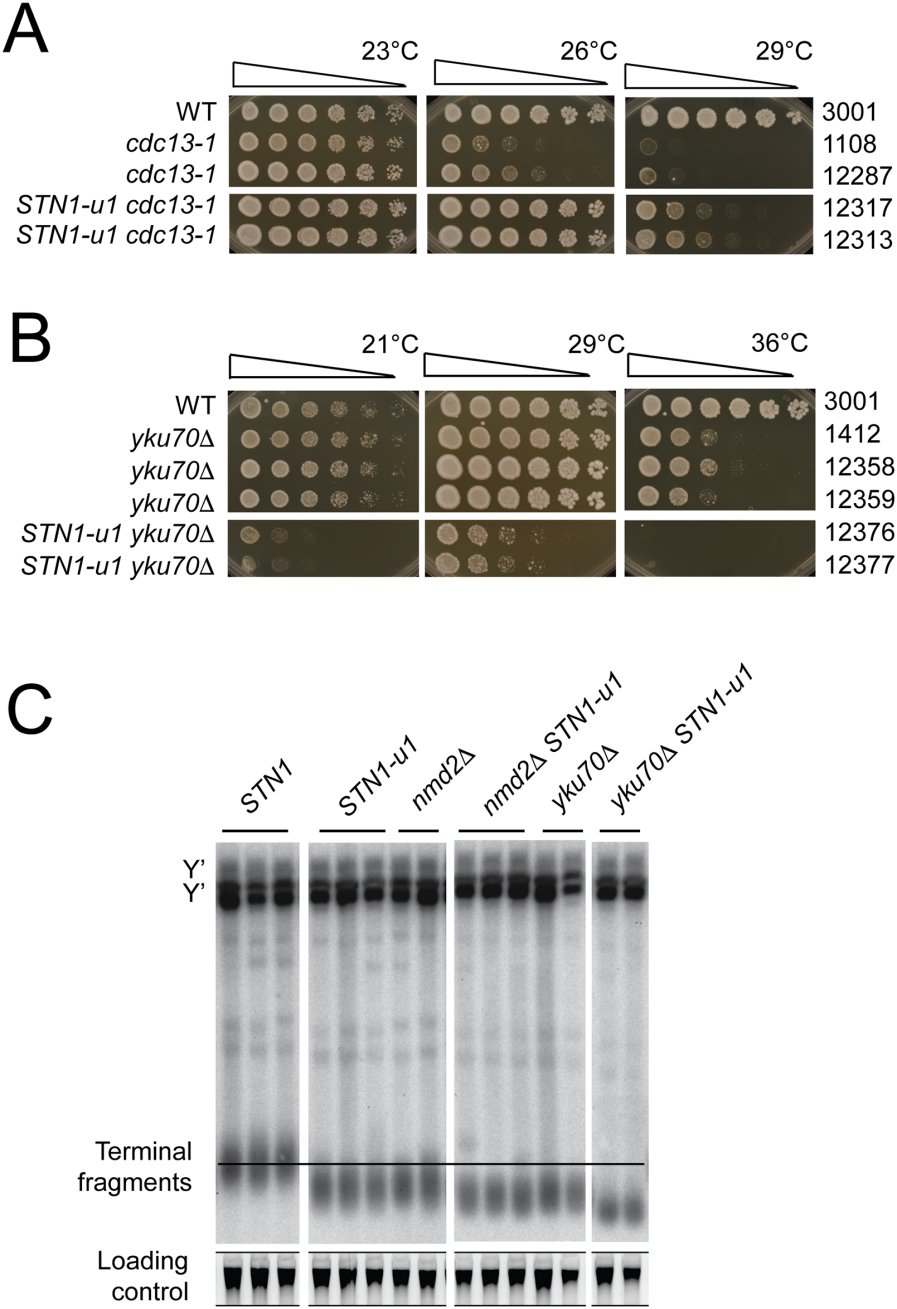
*STN1-u1* improves fitness of *cdc13-1* cells, decreases fitness of *yku70Δ* cells and causes short telomeres. A and B) Saturated cultures of the indicated strains were serially diluted, 5 fold, and spotted onto YEPD solid media and incubated for two days at indicated temperatures before being photographed. C) Genomic DNA was isolated from the yeast strains indicated, and telomere structures were analyzed by Southern blotting using a Y′ and TG probe, using SYBR Safe as a loading control, as previously described [26]. The image is cropped from a single membrane.

Interestingly, mild overexpression of *STN1*, due to a single copy (centromeric) plasmid containing *STN1* genomic DNA, suppressed temperature sensitive (ts), telomere-defective, *cdc13-1* cells and was the first reported connection between *STN1* and telomeres [14]. Therefore, it seemed likely that the *STN1* oORF affected fitness of *cdc13-1* cells. To test this *STN1-u1* was combined with *cdc13-1*. Interestingly, growth of *cdc13-1* cells at 26°C and 29°C was dramatically improved by *STN1-u1*, demonstrating that the *STN1* oORF acts to reduce the fitness of telomere defective *cdc13-1* cells (Fig 2A). The strong effect of *STN1-u1* on fitness of *cdc13-1* cells also allowed us to test whether *STN1-u1* was dominant or recessive. Interestingly, although perhaps as to be expected given that *STN1-u1* increases expression, *STN1-u1* is dominant over the wild type *STN1* allele in diploid *cdc13-1/cdc13-1* cells (S1 Fig).

To test the effects of the *STN1* oORF in the context of other telomere defects *STN1-u1* was combined with *yku70Δ*. *yku70Δ* cells have short telomeres and are temperature sensitive due to an accumulation of single stranded DNA near telomeres at higher temperatures [23]. Mild overexpression of *STN1*, using a low copy centromeric vector, has previously been shown to reduce fitness of *yku70Δ* cells at 36°C [24]. Interestingly, *STN1-u1* strongly reduced growth of *yku70Δ* cells at all temperatures tested (Fig 2B). The strong negative effect of *STN1-u1* on *yku70Δ* cell growth across the range of temperatures is unusual. For comparison, *nmdΔ* mutations, which also increase Stn1 levels, only inhibit *yku70Δ* growth at high temperatures [24].

Increased Stn1 levels, due to plasmid-induced overexpression or inactivation of the nonsense mediated mRNA decay pathway have been reported to reduce telomere length [12, 25]. Consistent with these results we observed that telomeres of *STN1-u1* cells were as short as those of *nmd2Δ* mutants (Fig 2C). Interestingly, a further reduction in telomere length was observed when *STN1-u1* was combined with *nmd2Δ* or *yku70Δ* mutations (Fig 2C). This data suggests that Nmd2 and Yku70 act at least somewhat independently of Stn1 levels to affect telomere length. In conclusion, high levels of Stn1 caused by *STN1-u1* strongly reduce telomere length.

### The NMD pathway and Tma20/Tma22 reduce levels of Stn1 via the oORF

The *STN1* oORF affects Stn1 levels and has at least three telomere related phenotypes, including strong suppression of *cdc13-1*. Published genome-wide *cdc13-1* suppressor analyses identified *nam7Δ*, *nmd2Δ* and *upf3Δ*, affecting the three central components of nonsense mediated decay, and *tma20Δ* and *tma22Δ* as similarly strong suppressors of *cdc13-1* (S2 Fig) [24]. *nmdΔ* mutations suppress *cdc13-1* principally by increasing Stn1 levels [22, 24]. *TMA20* and *TMA22* encode homologues of Drosophila/Human heterodimeric translation re-initiation factors MCT-1^Tma20^ and DENR^Tma22^ (S2 Fig), but how they affect *cdc13-1* cell fitness is unclear [27, 28]. MCT-1^Tma20^ and DENR^Tma22^ have been shown to promote expression of genes with uORFs [29, 30]. Tma20 and Tma22 were therefore plausible candidates to interact with the *STN1* uORF or oORF to affect Stn1 expression.

**Fig 3:**
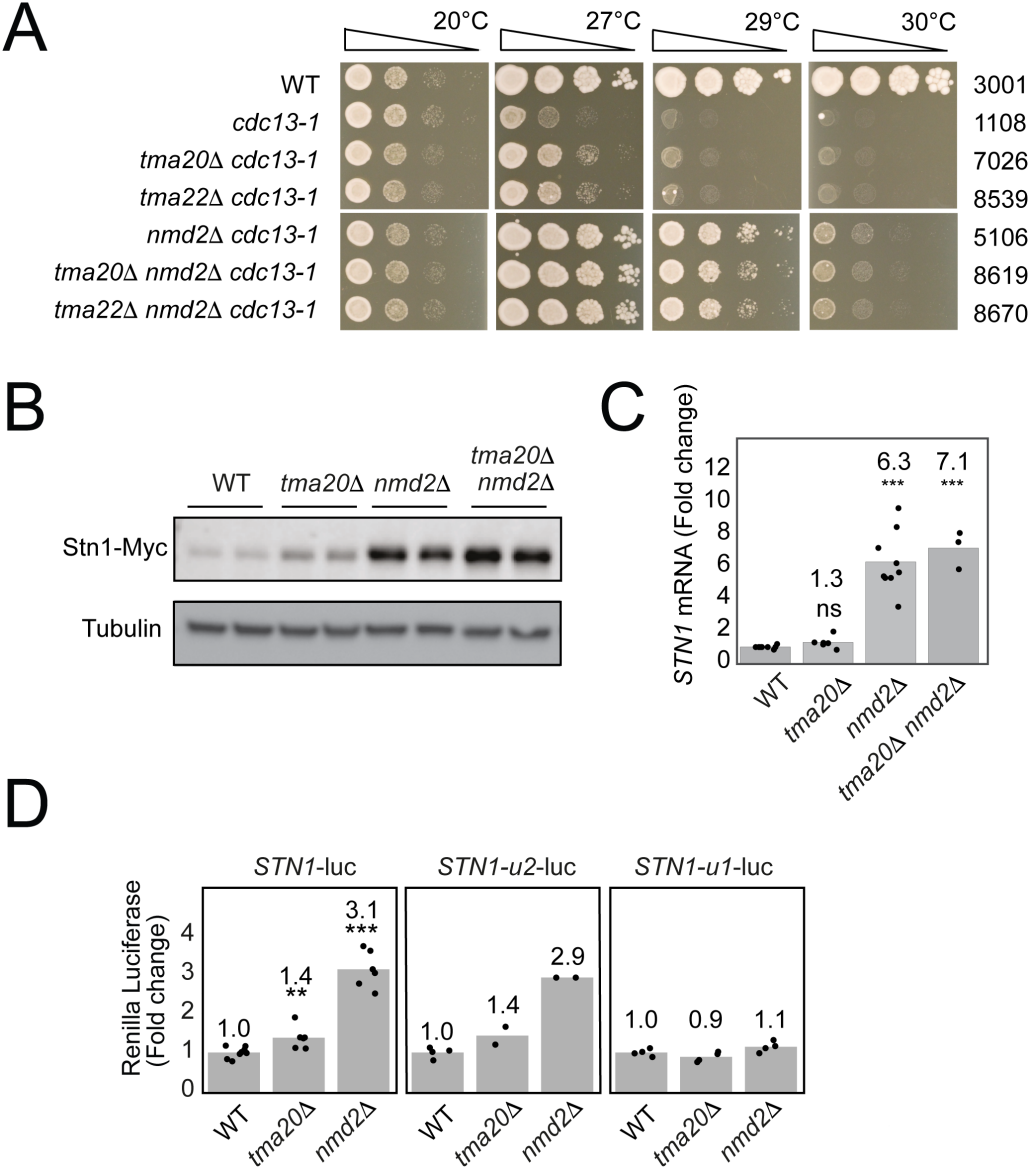
Nonsense mediated decay and translation re-initiation factor, Tma20, reduce levels of Stn1 via the oORF. A) Saturated cultures of indicated strains were serially diluted, 5-fold, and spotted onto YEPD solid media and incubated for two days at the indicated temperatures before being photographed. B) Western blot analysis of Stn1-Myc and tubulin levels. C) *STN1* transcript levels were measured and normalized as in Fig 1E. D). Dual luciferase reporter plasmids were used, as in Fig 1B, to measure the effects of *tma20Δ* and *nmd2Δ* on *STN1* URS activity. In each panel a different *STN1* URS construct was tested. The points on the graph represent independent measurements. *P* values were calculated using an unpaired t-test (**) *P* < 0.01, (***) *P* < 0.001.

To confirm that Tma20/Tma22 affected *cdc13-1* fitness similarly to the NMD pathway, the interactions between *tma20Δ*, *tma22Δ* or *nmd2Δ* and *cdc13-1* were tested by spot tests. By this assay, growth of *cdc13-1* cells was indeed improved by *tma20Δ* and *tma22Δ* but the effects were much less than the effect of *nmd2Δ* (Fig 3A). There was no difference in the fitness of *cdc13-1 tma20Δ*, *cdc13-1 tma22Δ* or *cdc13-1 tma20Δ tma22Δ* cells, in agreement with data suggesting that Tma20 and Tma22 function as a heterodimer, similarly to MCT-1^Tma20^ and DENR^Tma22^ [27, 28] (S2 Fig). *tma20Δ* or *tma22Δ* did not further improve fitness of *cdc13-1 nmd2Δ* cells (Fig 3A), suggesting that Tma20/Tma22 affect fitness of *cdc13-1* cells by a similar mechanism to the NMD pathway. Overall these data are consistent with the hypothesis that like Nmd2, Tma20 and Tma22 affect fitness of *cdc13-1* cells by decreasing levels of Stn1.

One hypothesis to explain why *tma20Δ* had weaker effects than *nmd2Δ* on *cdc13-1* cell fitness was that higher levels of Stn1 better suppressed *cdc13-1*, and that *tma20Δ* increased Stn1 levels less than *nmd2Δ*. To measure the comparative effects of *tma20Δ* and *nmd2Δ* on Stn1 expression, western blots were performed. As previously reported Stn1 levels were several fold higher in *nmd2Δ* cells [22], and as predicted, slightly higher in *tma20Δ* cells (Fig 3B and S3 Fig). *nmd2Δ tma20Δ* cells had marginally higher Stn1 levels than *nmd2Δ* single mutants, suggesting that Nmd2 and Tma20 affect Stn1 levels independently (Fig 3B).

It is known that NMD affects Stn1 levels by affecting RNA abundance [17], and since Tma20 and Tma22 homologs affect translation initiation [29, 30], it seemed plausible that Tma20/Tma22 instead affected *STN1* translation. Consistent with this hypothesis, *tma20Δ* did not significantly increase *STN1* transcript levels (Fig 3C), whereas, *nmd2Δ* increased transcript (and protein levels) about 6-fold (Fig 3B and 3C, S3 Fig). These data show that Tma20 affects Stn1 protein levels without strongly affecting transcript levels.

To test if Nmd2 and Tma20 affect Stn1 expression via the uORF or oORF, luciferase assays were used. Expression driven by *STN1*, *STN1-u2* and *STN1-u1* URSs in wild type, *tma20Δ* and *nmd2Δ* backgrounds was measured. Consistent with the western blot analysis of strains with integrated alleles there was a small increase in expression of *STN1-luc* in *tma20Δ* cells, and a larger increase in *nmd2Δ* cells (Fig 3B and 3D). The *STN1-u2-luc* construct, lacking the most upstream uORF, showed a similar pattern of expression to the native *STN1* construct in wild type, *tma20Δ* and *nmd2Δ* cells (Fig 3D). These results suggest that Nmd2 and Tma20 do not reduce *STN1* expression via the *STN1* uORF. In contrast, however, *STN1-u1-luc*, lacking the oORF, showed a very different pattern, with very similar levels of gene expression being observed in wild type, *tma20Δ* and *nmd2Δ* cells (Fig 3D and S3 Fig). These data indicate that both Tma20 and Nmd2 reduce Stn1 expression principally via the oORF. Consistent with these plasmid-based luciferase assays, we did not observe any further increase in the levels of Stn1-Myc when *nmd2Δ* was combined with *STN1-u1* (S4 Fig). Furthermore, no notable changes in *STN1* transcript levels were observed between *nmd2Δ* and *nmd2Δ STN1-u1* cells (S4 Fig). Overall, these data support the view that Tma20 and Nmd2 affect Stn1 levels principally via the oORF and that their effects on Stn1 levels can explain their effects on fitness of *cdc13-1* telomere-defective cells.

### Tma20 dependent regulation of Stn1 levels requires the oORF stop codon

**Fig 4:**
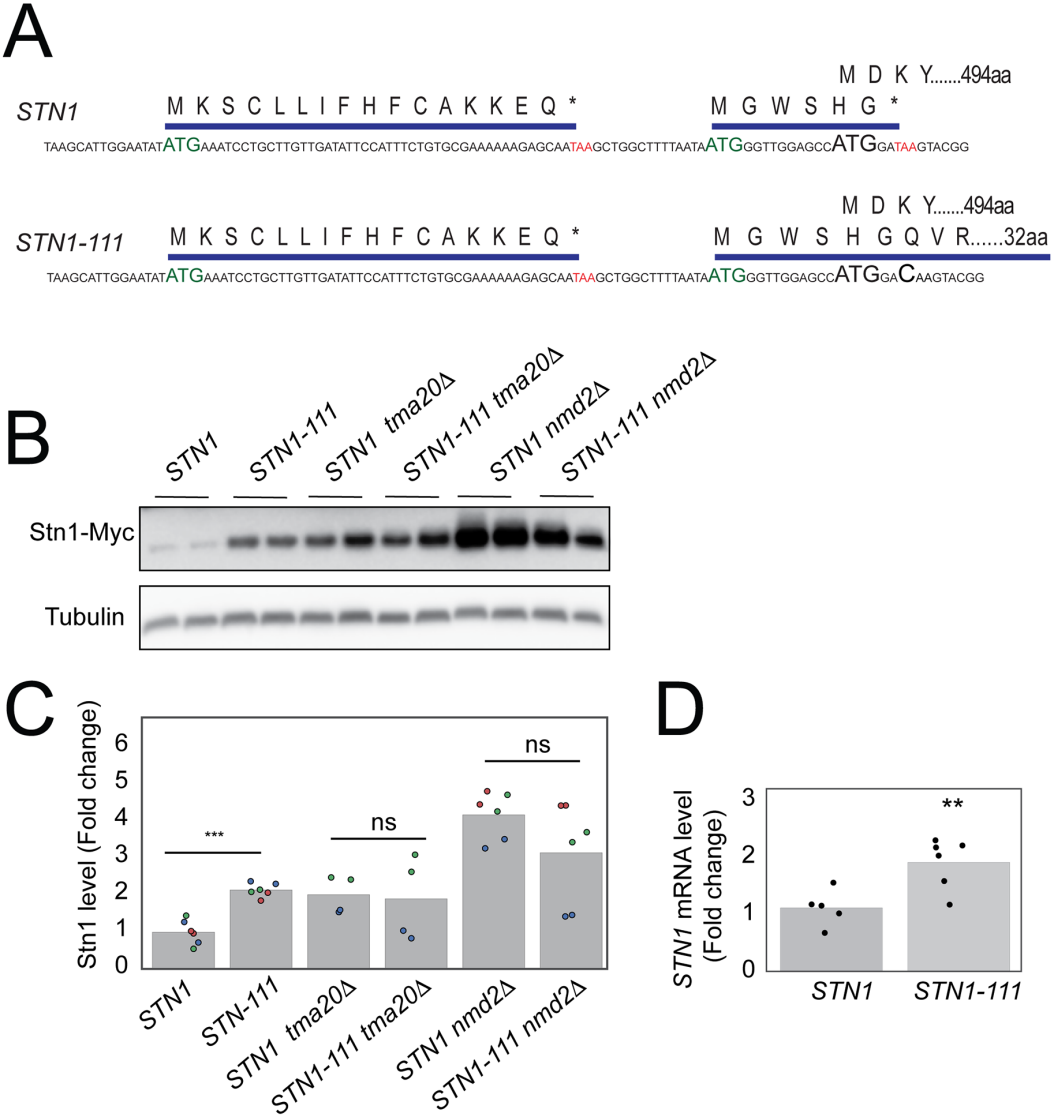
The *STN1* oORF termination codon affects Stn1 levels. A) *STN1-111* is a point mutation in the termination codon of the natural oORF, extending the oORF from 6 to 32 aa. B) Western blot analysis of Stn1-Myc and tubulin levels. C) Quantification of western blot in B and S9 Fig. The points on the graph represent independent measurements, and points in the same colour are from the same western blot. D) *STN1* transcript levels were measured as in Fig 1E (**) *P* < 0.01.

The *STN1* oORF terminates just 2 nucleotides after the main *STN1* ATG and we wondered if this proximity contributed to the effectiveness of the oORF in reducing gene expression. To test this notion, a point mutation in the oORF termination codon was introduced, thus creating *STN1-111*, which increases the length of the oORF from 6 to 32 amino acids (Fig 4A). Interestingly, *STN1-111* increased levels of Stn1 (Fig 4B and 4C), suggesting that either the short length of the natural *STN1* oORF, or the proximity of the oORF stop codon to the *STN1* CDS initiation codon, contributes to the effectiveness of the *STN1* oORF in reducing gene expression. Furthermore, the levels of Stn1 are similar in *STN1-111*, *tma20Δ* and *STN1-111 tma20Δ* cells, suggesting that Tma20 decreases *STN1* expression by a mechanism that is dependent on the context of the native oORF termination codon (Fig 4C and S4 Fig).

The effects of Nmd2 on expression of *STN1-111* were also examined. While *nmd2Δ* increased *STN1-111* mRNA and protein levels the effect was proportionately less than on wild type *STN1* mRNA and protein levels (Fig 4B and 4C and S4 Fig). These data suggest that the *STN1-111* transcript is a weaker target for the NMD pathway than the *STN1* transcript and explain why the *STN1-111* transcript is more abundant than *STN1* (Fig 4D).

To explore how Tma20, Nmd2 and the Stn1 oORF interact to affect fitness of telomere-defective stains we combined *nmd2Δ* and *tma20Δ* with *STN1-u1* and *STN1-111* in a *cdc13-1* background. Consistent with the idea that Tma20 reduces fitness of *cdc13-1* cells via the *STN1* oORF, *tma20Δ* does not further increase the fitness of *cdc13-1 STN1-u1* or *cdc13-1 STN1-111* cells (S5 Fig). However, the correlation between Stn1 expression levels in *nmd2Δ* and *STN1-u1* cells and the effect of these mutations on *cdc13-1* fitness was less clear. *nmd2Δ* increased Stn1 less than *STN1-u1* but better improved the fitness of *cdc13-1* cells (S4 and S5 Figs). It is known that NMD affects levels of hundreds of transcripts, including many encoding telomerase components and regulators, such as Ten1 [17]. We rationalized that *cdc13-1 nmd2Δ* mutants may be fitter than *cdc13-1 STN1-u1* mutants because *nmd2Δ* causes overexpression of both Stn1 and Ten1 while *STN1-u1* only affects Stn1 levels [22]. Consistent with this hypothesis, plasmid driven overexpression of Ten1 further improved fitness of *cdc13-1 STN1-u1* cells (with already very high levels of Stn1 overexpression) while having barely any effect on *cdc13-1* cells (S5 Fig). We conclude that suppression of *cdc13-1* telomere defects can be improved by coordinated overexpression of its two partner proteins, Stn1 and Ten1.

### Human Stn1 has oORFs that reduce expression

**Fig 5:**
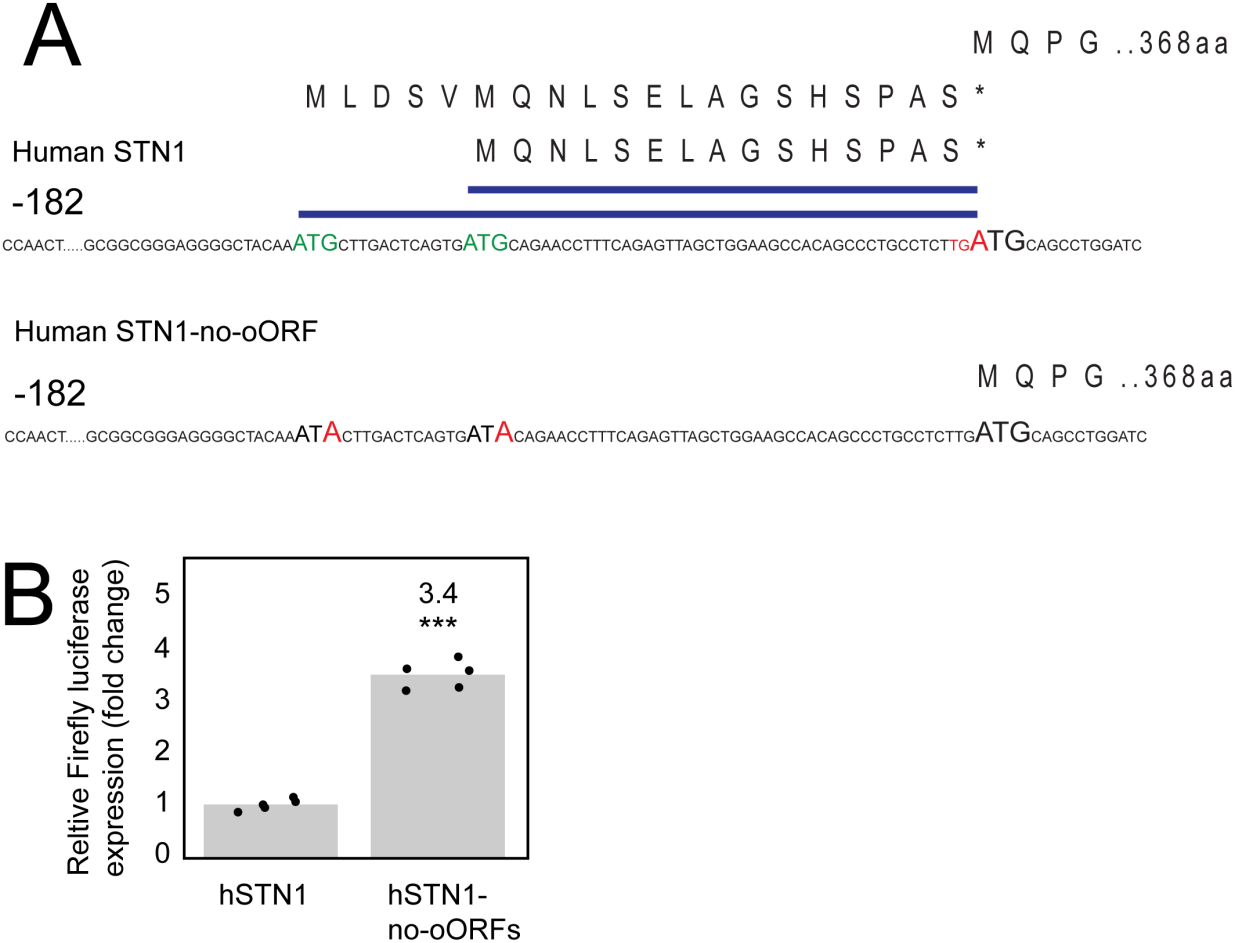
Human Stn1 has two oORFs that reduce expression. A) Sequences of human *STN1* transcript leader indicating the positions of the oORFs. B) Dual luciferase assay of *STN1* driven URS expression in human cells. HCT116 cells were transfected with pGL3 expressing FLuc under the control of *STN1* or *STN1-no-oORF* TLs. Co-transfected pRL-TK expressing RLuc was used as a normalization control. Values are normalized to the loading control and calculated relative to *STN1* as in Fig 1B. Each point represents a measurement from an independent transfection. *P* values were calculated using an unpaired t-test (***) *P* < 0.001.

It is clear that the yeast *STN1* oORF plays an important role in maintaining low levels of Stn1, which in turn contributes to critical telomere functions, such as capping and length control. Given that Stn1 has conserved functions in human cells, we wondered if an oORF was also found in the human *STN1* transcript leader (TL). Indeed, analysis of the human *STN1* revealed that there are two, encoding overlapping 15 and 19 amino acid oORFs, sharing a stop codon (Fig 5A). The third nucleotide of the ORF stop codons is the first nucleotide of the *STN1* CDS (Fig 5A). To test whether the oORFs of *STN1* reduce expression in human cells *STN1* URS activity was measured using dual luciferase assays. Point mutations introduced into each of the oORF initiation codons, *STN1-no-oORF*, increased expression 3.4 fold in human cells. Therefore, human *STN1*, like yeast *STN1*, is regulated by overlapping open reading frames that strongly reduce *STN1* expression.

### Natural selection against oORFs is stronger than against than uORFs

**Fig 6:**
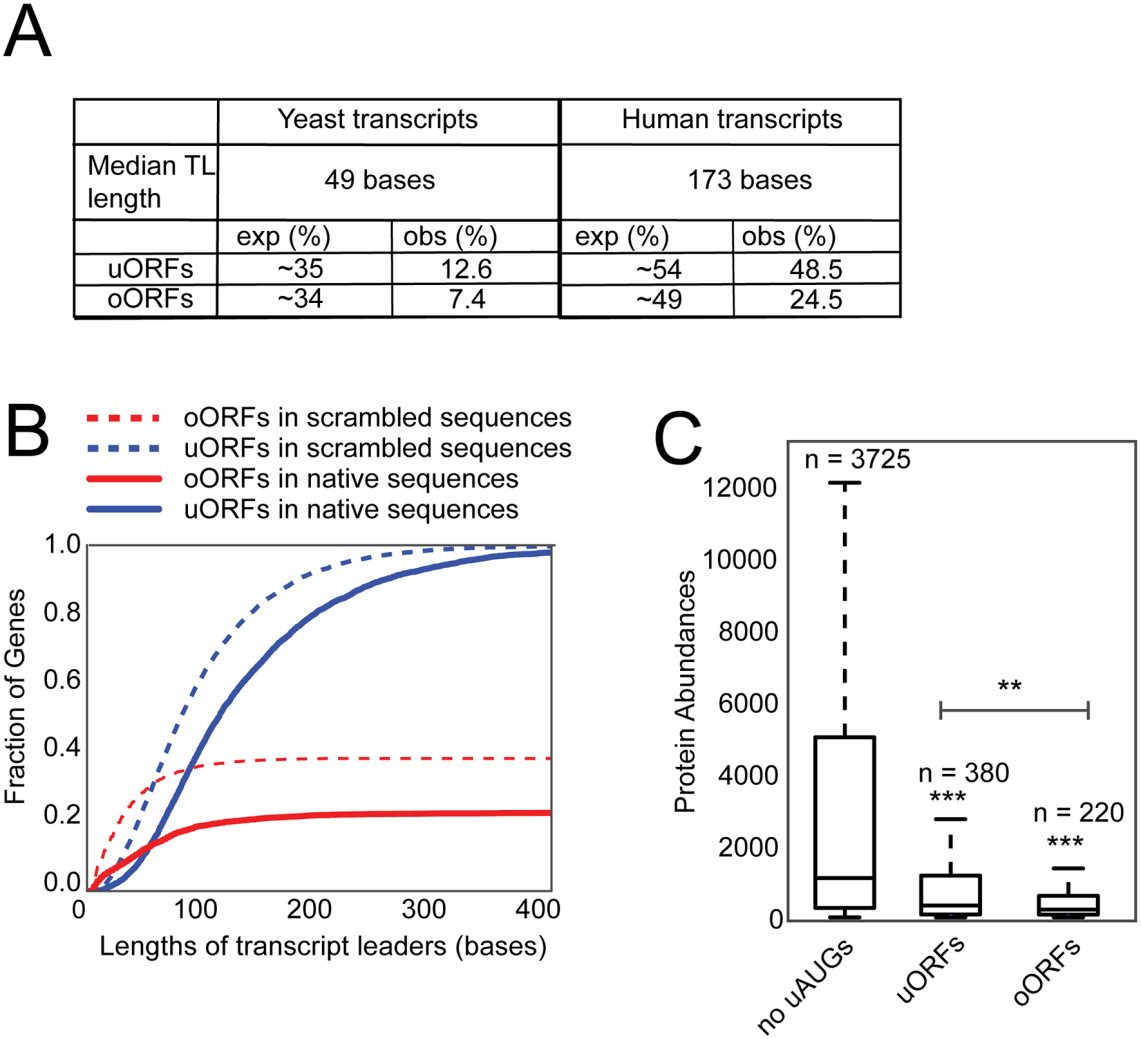
oORFs are more selected against than uORFs. A) The percentage of protein coding genes in yeast and human cells that contain uORFs or oORFs. The expected fraction was calculated from a single randomization of each TL. Several independent randomizations were performed with very similar results. B) The relationship between transcript leader lengths and observed and expected numbers of uORFs (3 reading frames) and oORFs (2 reading frames) in yeast URSs C) Box plots indicating the relationship between protein abundance, uORFs and oORFs for yeast genes. Genes were grouped into those with uORFs, or ORFs, for simplicity genes with both are not plotted. Protein abundance measurements are taken from [31]. The box indicates 25^th^ and 75^th^ percentiles, with the difference between these two being the interquartile range (IQR). The upper whisker represents the largest data point less than 1.5 x IQR. Similarly, the lower whisker represents the smallest data point greater than 1.5 x IQR. Any data points outside of this range are considered to be outliers and not indicated. P values are calculated using an unpaired t-test. (**) *P* < 0.01 (***) *P* < 0.001.

The yeast *STN1* oORF more strongly reduces gene expression than the *STN1* uORF and therefore it was possible that this is a general phenomenon. To systematically explore the effects of uORFs and oORFs in other contexts, yeast and human genomes were analyzed to calculate the fraction of transcripts that contain uORF and oORFs. To infer the effects of natural selection these numbers were compared to the number of uORFs and oORFs calculated to occur in individually scrambled TL sequences (Fig 6A). In yeast the proportion of transcripts with uORFs or oORFs observed was far less than expected based on analysis of scrambled sequences (12.6% observed vs 35% expected for uORFs, 7.4% vs 34% for oORFs). In humans the proportion of transcripts with uORFs or oORFs was also less than expected (48.5% vs 54% for uORFs, 24.5% vs 49% for oORFs). uORFs and oORFs are more common in human than yeast TLs, presumably because human TL sequences, median length 173 nucleotides, are longer than yeast, median length 49 nucleotides. However, the relationship between TL length and the likelihood of uORFs or oORFs being present was unclear. To explore this relationship, the theoretical number of uORFs and oORFs found with increasing length of yeast TL sequences was calculated. As expected uORFs and oORFs increase in frequency as TL length increases, but the observed frequency was always less than expected from analysis of randomized sequence, presumably reflecting natural selection (Fig 6B). Importantly, at TL lengths longer than 50 bases, or so, there are always proportionately fewer oORFs than uORFs (S6 Fig), consistent with the idea that oORFs have been more strongly selected against than uORFs.

Finally, in yeast there is a significant reduction in protein abundance associated with genes that encode oORFs, in comparison with those that encode uORFs (Fig 6C). Together these data are consistent with the view that, on average, oORFs more strongly reduce gene expression than uORFs.

## Discussion

We have shown that levels of Stn1 are dramatically reduced by upstream oORFs. In yeast, *STN1* contains both a uORF and an oORF but the latter is a far more potent inhibitor of gene expression. The *STN1* oORF reduces gene expression by at least two routes. First, the *STN1* oORF targets the transcript for degradation by NMD, and second, it reduces translation efficiency in a Tma20/Tma22 dependent manner. Reducing the efficacy of the *STN1* oORF by mutating its initiation codon, its stop codon, or by inactivating *NMD2* or *TMA20* results in a range of different telomere related phenotypes showing how important the Stn1 oORF is to telomere function.

In yeast, it is clear that low Stn1 levels are critical for normal telomere function [12]. Furthermore, it has been suggested that when overproduced Stn1 performs additional functions, is recruited to non-telomeric sites and overrides the S-phase checkpoint [15]. We now show the yeast *STN1* oORF is key to maintaining low levels and appropriate functions. Analogously, in human cells, where STN1 has been shown to have a role in replication of non-telomeric DNA, perhaps oORF regulation affects the balance between telomeric and non-telomeric roles of Stn1 [32, 33].

The NMD pathway was originally defined as targeting mRNAs that contained premature termination codons (PTCs), but the list of NMD targets is expanding. It now includes mRNAs that contain uORFs, long 3’UTRs, frameshifts, unspliced introns, aberrant transcript isoforms and otherwise normal transcripts with low translation efficiencies due to out-of-frame translation or lower than average codon optimality [17, 34, 35]. In this case we have established that *STN1* mRNA targeting by NMD depends on both the start and stop codons of the oORF, suggesting that the short length of the oORF and/or the close proximity of the oORF termination to the *STN1* CDS initiation are important for NMD to target the *STN1* transcript.

In *Drosophila* and mammalian cell-based experiments MCT-1^Tma20^/DENR^Tma22^ contributed to increased expression of transcripts that contain uORFs, whereas we found that Tma20/Tma22 reduced expression of *STN1* via the oORF [29, 30]. Biochemical experiments show that MCT-1^Tma20^/DENR^Tma22^ promote the dissociation of the 40S ribosome subunit following translation termination [36]. One hypothesis that may explain the effects of Tma20/Tma22 on *STN1* expression in yeast is that Tma20/Tma22 reduce translation re-initiation by promoting 40S ribosome subunit dissociation (S7 Fig). This hypothesis is supported by the finding that Tma20/Tma22-dependant regulation of Stn1 levels requires the *STN1* oORF stop codon to be in close proximity to the *STN1* CDS initiation codon. Furthermore, MCT-1^Tma20^/DENR^Tma22^ have been shown to affect translation re-initiation on some bicistronic viral transcripts [37].

It has become increasingly clear that uORFs are efficiently translated and associated with lower levels gene expression [19, 20]. Mutations in uORF initiation and termination codons have been linked to human malignancies [38] and a mutation introducing a uORF into the TL of CDKN2A causes familial predisposition to melanoma [39]. In specific cases uORFs have been shown to act as regulatory mechanisms to control gene expression under conditions of stress. The best studied examples are *ATF4* and *GCN4* which encode homologous transcription factors in human and yeast cells [40, 41]. The levels of each are regulated by uORFs and oORFs which facilitate translational upregulation in response to nutrient stress [40, 41]. In a more general sense, uORFs have been suggested to permit the preferential translation of specific transcripts following DNA damage [42]. The *STN1* oORF provides, in principle, a powerful mechanism to rapidly increase Stn1 levels. It was recently shown that growth at high temperature increases yeast Stn1 levels [43], and it would be interesting to determine whether the *STN1* oORF contributes to this type of upregulation.

The *STN1* oORF clearly provides a powerful mechanism to reduce Stn1 levels appropriately, to positively affect telomere function. More generally it seems that oORFs are more potent than uORFs at inhibiting gene expression. oORFs may therefore be part of a powerful mechanism of reducing protein levels across biology.

## Methods

### Yeast strain growth and transformation

Standard procedures for yeast culture, mating and tetrad dissection were followed. Strain genotypes are in S1 Table. W303 strains were used and YEPD was supplemented with adenine (75 mg/l). Gene deletions were performed using one-step PCR to replace *TMA22* with a marker and confirmed by PCR. *STN1* point mutations were introduced into the genome using integrative plasmids based on pRS406. These strains contained duplicated copies of the *STN1-PDC2* URS separated by a *URA3* gene. The URS adjacent to *STN1* contained uORF point mutations and the URS adjacent to *PDC2* did not (S8 Fig). As a control, the same construct without mutations was also integrated. Primers and plasmids are listed in S2 and S3 Tables.

### Growth assays

A pool of colonies (>10) were grown in 2 mL liquid YEPD or SC media overnight at 23°C. Serial dilutions, as indicated in legends, were made in water and spotted onto round or rectangular agar plates using a replica plating device. Plates were incubated for indicated number of days at the indicated temperatures before being photographed.

### Protein extraction and western blots

Protein extractions and western blots were performed essentially as described previously [44]. 10 mL mid log phase cells were washed twice with 2 mL of 20% TCA and re-suspended in 100 μL of 20% trichloroacetic acid (TCA). Cell pellets were frozen at -80°C. Once thawed 100 μL glass beads were added to the pellets and cells mechanically lysed using a Precellys (2x 15s 6,500rpm). 200 μL of 5% TCA was added and samples briefly vortexed. Samples were centrifuged at 13,000 rpm for 10 minutes at 4°C and pellets re-suspended in 100 μL Laemmli loading buffer (Bio-Rad) with 5% β-mercaptoethanol. 20 μL 1M Tris was added to samples to neutralise the pH. Samples were boiled for 3 minutes, centrifuged at 13,000 rpm for 10 minutes and the supernatant transferred to a clean tube. 10 μL of protein extracts were loaded onto a gradient (4–15%) precast gel (Bio-Rad Mini-Protean TGX) and run for 90 minutes at 100V in Tris/Glycine/SDS running buffer (Bio-Rad). Proteins were blotted, for 30 minutes, onto nitrocellulose membranes using the Trans-Blot Turbo Transfer System (Bio-Rad) according to the manufacturer’s protocol. Anti-C-Myc (9E10 (1 in 2000 in 1% milk) from Abcam) was used to detect Stn1-MYC and anti-tubulin (1 in 2000) antibodies from Keith Gull, Oxford, UK, was used to detect Tubulin as a loading control. Proteins were detected using Thermo Scientific SuperSignal West Pico Chemiluminescent Substrate according to the manufacturer’s instructions and imaged on a G-box imager (Syngene).

### Quantitative RT-PCR

RNA purification and RT-PCR was carried out as described previously [45]. RNA was purified using the RNEasy Mini Kit (QIAGEN, 74104) and by DNase I digestion (Invitrogen, 18068–015). Quantitative RT-PCR was carried out using the Superscript III Platinum SYBR Green One-Step qRT-PCR kit (Invitrogen, 11736–059). 2 μL RNA sample (80 ng/μL) was added to 8 μL of the reaction mix (10 μM F primer (0.2 μL), 10 μM R primer (0.2 μL), Superscript III Platinum Taq Mix (0.2 μL), 2x SYBR Green Reaction Mix (5 μL), ROX reference dye (0.2 μL), DEPC-treated water (2.2 μL)) in a 96 well plate. An ABI Systems StepOnePlus thermal cycler was used (1 cycle: 50°C for 3 minutes, 1 cycle: 95°C for 5 minutes, 40s cycle: 95°C for 15 seconds then 60°C for 30 seconds, 1 cycle: 40°C for 1 minute). RNA samples were normalized relative to the *BUD6* loading control.

### Analysis of telomere length

Southern blot analysis was used to assess telomere length as previously described [46]. Genomic DNA was extracted, digested with XhoI and run overnight on a 1% agarose gel at 1 V/cm. Southern transfer was performed using a BioRad Vacuum Blotter according to manufacturer’s instructions. Probe labelling, hybridization and washing were performed according to the DIG High Prime DNA Labelling and Detection Starter Kit II (Roche) instructions. A probe that annealed to the Y’ elements and TG telomere repeats was made using DNA from a plasmid (pDL987) that contained 120 bp of TG repeats and 752 bp of the upstream Y’ element from telomere VIII-R. Loading control pictures were taken from each gel, before transfer, using SYBR Safe to stain the total DNA.

### Mammalian cell culture and transfection and dual luciferase assays

HCT116 F/F CTC1 cells (obtained from Carolyn Price and containing loxP sites at the CTC1 locus) were maintained in McCoys 5a media (supplemented with foetal bovine serum (10%), PenStrepGlut (Corning) and puromycin (100 ng/μL)) under 5% CO_2_ in a humidified incubator. 1 μg plasmid DNA was transfected into 1 x 10^6^ cells using a Nucleofector (Lonza) according to manufacturer’s protocol and incubated for 24 hours in a 6 well plate. Dual-Luciferase Reporter Assay System (Promega) was used to measure luciferase expressed from mammalian cells according to manufacturer’s protocol using the active lysis by scraping method.

### Luciferase assays—Yeast

Plasmids were designed to quantify gene expression *in vivo* using two different reporter genes, encoding Renilla and Firefly luciferases. Firefly luciferase was fused to a constitutive promoter, *PGK1*, to act as a ‘loading control’. Renilla luciferase was fused with *STN1* URSs, which encompassed the promoter and TL. To help ensure that expression of the test URS was unaffected by the loading control the genes encoding Renilla and Firefly luciferase were fused to *ADH1* and *CYC1* terminators respectively, and orientated in opposite orientations. 2 mL yeast cultures were grown to saturation overnight in—LEU media in a rotating wheel in an incubator at 30°C. From this a fresh 2 mL culture was inoculated with 100 μL saturated cells and rotated at 30°C until an OD_600_ of 0.6–0.8 was achieved (6 hours). Cultures were harvested and re-suspended in 90 μL of passive Lysis buffer from Dual-Luciferase Reporter Assay System (Promega). 10 μL of cells suspension were then added to a well of a 96-well white plate. A PolarStar (Omega) plate reader was programmed to dispense 50 μL of LAR II (Promega), shake for 5 seconds, record the luminescence signal (4 readings with 0.5 second interval times), dispense 50 μL Stop & Glo Reagent (Promega), shake for 5 seconds, and again record the luminescence signal (4 readings with 0.5 second interval times). The mean of the 4 readings that were recorded was used as the final measurement.

### Bioinformatic analyses of uORFs and oORFs

For yeast, the sequences of all genes, plus 1000 bases upstream, were retrieved from YeastMine [47]. TL lengths were obtained from [48], using the longest isoform reported, or if unavailable from [49]. The locations of uORF and oORFs were determined computationally and are shown in S1 File. The Python scripts to identify the uORFs and oORFs within the transcript leader sequences can be found on Github (https://github.com/vickytorrance/uAUGs). To infer the effects of natural selection the TL sequences were individually randomized once and the number of uORFs and oORFs in randomized sequences calculated. For human transcripts, cDNA and TL sequences were downloaded from Biomart (Ensembl), only considering ‘Protein coding gene’ transcripts and the presence of uORFs or oORFs calculated as for yeast (S2 File).

## Supporting information

S1 Fig *STN1-u1* is dominant. Saturated cultures of indicated genotypes were serially diluted, 5-fold, spotted onto YEPD solid media and incubated for two days at indicated temperatures before being photographed.
(TIF)

S2 Fig *TMA20* and *TMA22* encode conserved genes and have similar effects on the fitness of *cdc13-1* cells. A) *cdc13-1* or *CDC13* strains were combined with the yeast knockout collection and fitness (maximum doubling rate X maximum doubling potential) determined at 27°C [24]. Each spot corresponds to the position of a single gene deletion. *cdc13-1* suppressors (blue) or enhancers (red) are indicated. B) Domain organisation of Tma20^MCT-1^ and Tma22^DENR^ reproduced from [36]. Tma20^MCT-1^ contain a PseudoUridine synthase and Archaeosine transglycosylase domain (PUA). Tma22^DENR^ contains a SUI1 domain. C) Saturated cultures, of indicated genotypes, were serially diluted, 5 fold, spotted onto YEPD solid media and incubated for two days at indicated temperatures before being photographed.
(TIF)

S3 Fig Effects of *TMA20* and *NMD2* on *STN1* expression. A) Quantification of Fig 3B and S9 Fig. B) Data in Fig 3D but normalized relative to expression of *STN1* in WT cells. The points on the graph represent independent measurements, and are coloured according to the date that they were obtained. *P* values were calculated using an unpaired t-test (**) *P* < 0.01, (***) *P* < 0.001.
(TIF)

S4 Fig Effects of *NMD2* and *STN1* mutations on *STN1* expression. A) Western blot analysis of Stn1-Myc and Tubulin levels. The image is cropped from a single membrane. B) *STN1* transcript levels compared with data also shown in Figs 1E, 3C and 4D.
(TIF)

S5 Fig Effects of *TMA20*, *NMD2*, *STN1-u1*, *STN1-111* and *TEN1* overexpression on fitness of *cdc13-1* cells. A-C) Saturated cultures of indicated genotypes were serially diluted, 5 fold, and spotted onto YEPD (A, B) or—LEU (C) solid media and incubated at indicated temperatures before being photographed.
(TIF)

S6 Fig Relationship between transcript leader length and the fraction of uORFs and oORFs. For each TL length the fraction of uORFs or oORFs observed in native sequences over those in randomized sequences was calculated (using the data from Fig 6B).
(TIF)

S7 Fig Model of how Tma20/Tma22 affect translation re-initiation after oORF. Following translation termination at the ribosome stop codon the 60S subunit dissociates from the termination complex. Tma20/Tma22 then promotes the dissociation of the 40S from the mRNA thus preventing re-initiation. The idea that Tma20/Tma22 reduce translation re-initiation is also supported by evidence showing that interaction of MCT-1^Tma20^/DENR^Tma22^ with the 40S subunit is incompatible with the binding of the 60S ribosomal subunit to the 40S subunit [27].
(TIF)

S8 Fig Diagram of the *STN1-PDC2* locus. A) A map of the *STN1-PDC2* locus. B) To ensure that expression of the adjacent gene (*PDC2*) was unaffected by mutations, the sequence separating *STN1* and *PDC2* was duplicated and separated by *URA3*. This was achieved by transforming yeast strains with an integrative plasmid assembled from three PCR products, labelled A, B and C. Fragment A was amplified using WT genomic DNA. Fragment B was amplified from pFA6*URA3* (pDL1833). Fragment C was amplified from genomic DNA extracted from *STN1 (STN1-u1* (DLY 11871) and *STN1-u2* (DLY 11870) cells that contained additional unwanted point mutations in *STN1* CDSs, using primers designed to separate *STN1-u1* and *STN1-u2* from the mutations in the CDSs. Primers used for PCR are indicated by mXXXX.
(TIF)

S9 Fig Additional western blots. Blots providing additional data shown in Figs 1D, 4B and S3 Fig.
(TIF)

S1 Table Table of yeast strains used in this study. (DOCX)

S2 Table Table of primers used in this study. (DOCX)

S3 Table Table of plasmids used in this study. (DOCX)

S1 File Yeast upstream and overlapping open reading frames. The start column indicates the position of first base of the uAUG with respect to the first base of the initiation codon of the CDS. The stop column indicates the position of the third base of the uORF or oORF stop codon with respect to the first base of the initiation codon of the CDS.
(CSV)

S2 File Human upstream and overlapping open reading frames. The start column indicates the position of first base of the uAUG with respect to the first base of the initiation codon of the CDS. The stop column indicates the position of the third base of the uORF or oORF stop codon with respect to the first base of the initiation codon of the CDS.
(CSV)

S1 Dataset Numerical data plotted in graphs. (XLSX)
