# Supplementary figures and images for: Overlapping open reading frames strongly reduce human and yeast STN1 gene expression and affect telomere function
Source: PLoS Genet. 2018 Aug 1;14(8):e1007523. doi: 10.1371/journal.pgen.1007523 (PMC6089452; doi:10.1371/journal.pgen.1007523)

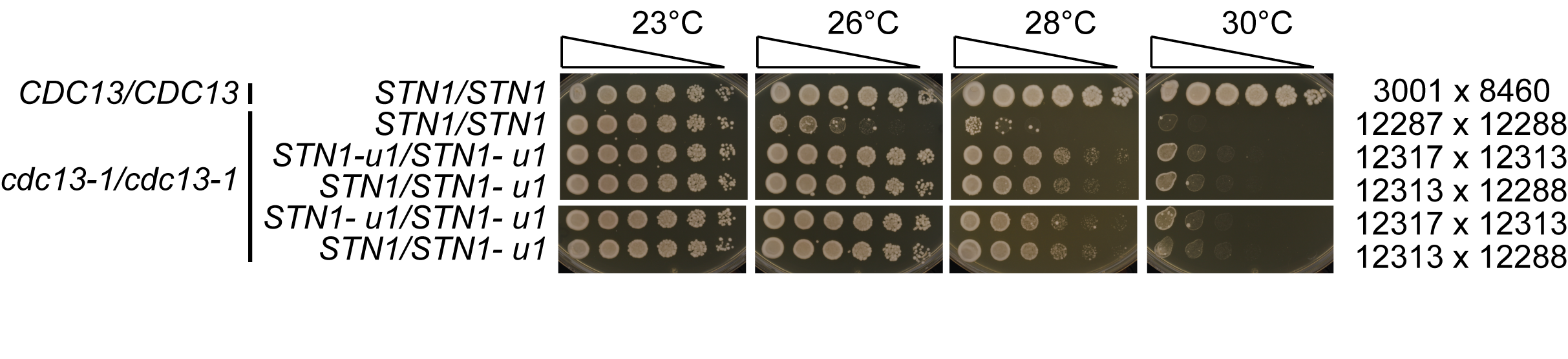

Supplement: S1 Fig — Saturated cultures of indicated genotypes were serially diluted, 5-fold, spotted onto YEPD solid media and incubated for two days at indicated temperatures before being photographed. (TIF) [file pgen.1007523.s001.tif]

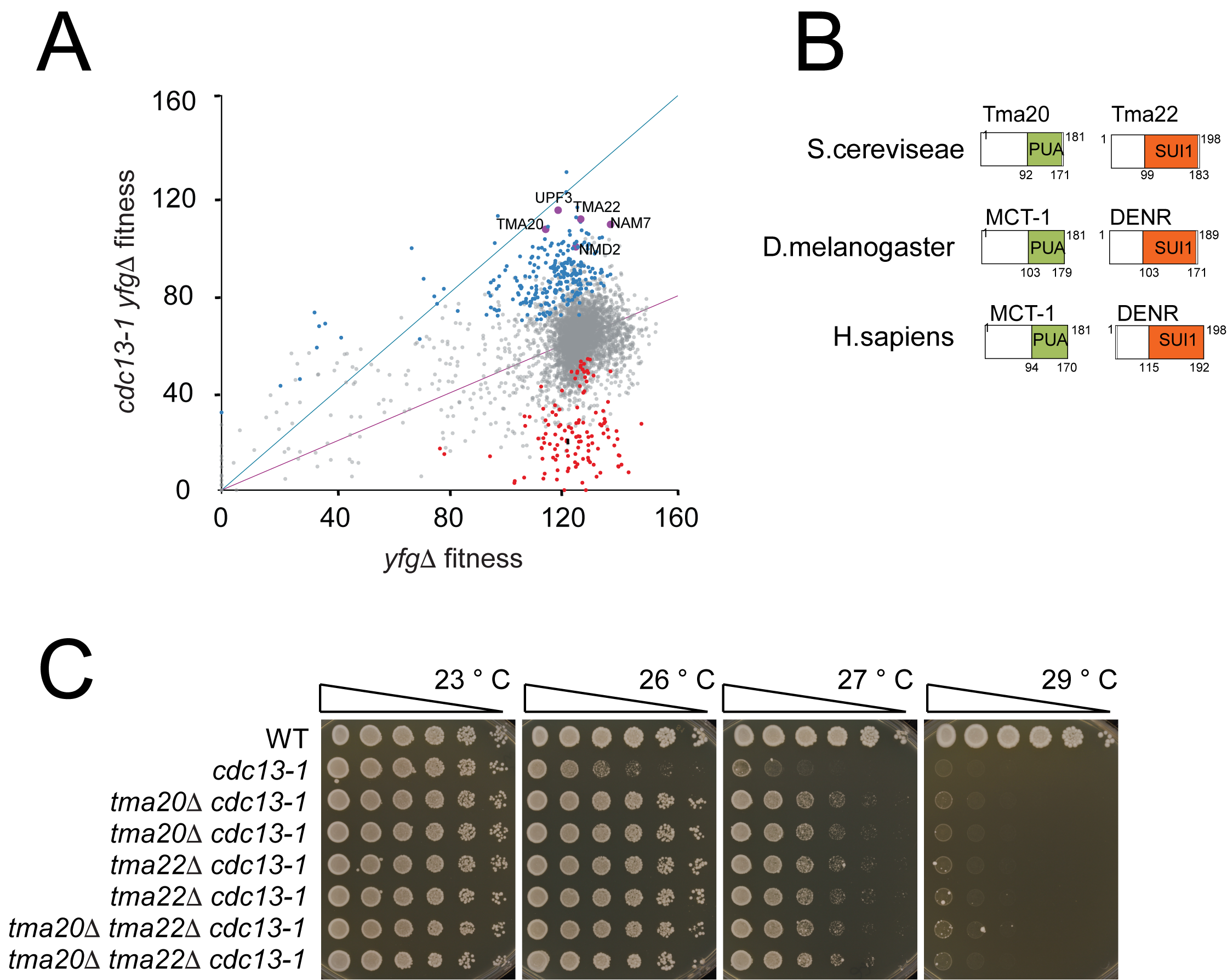

Supplement: S2 Fig — A) cdc13-1 or CDC13 strains were combined with the yeast knockout collection and fitness (maximum doubling rate X maximum doubling potential) determined at 27°C [24]. Each spot corresponds to the position of a single gene deletion. cdc13-1 suppressors (blue) or enhancers (red) are indicated. B) Domain organisation of Tma20MCT-1 and Tma22DENR reproduced from [36]. Tma20MCT-1 contain a PseudoUridine synthase and Archaeosine transglycosylase domain (PUA). Tma22DENR contains a SUI1 domain. C) Saturated cultures, of indicated genotypes, were serially diluted, 5 fold, spotted onto YEPD solid media and incubated for two days at indicated temperatures before being photographed. (TIF) [file pgen.1007523.s002.tif]

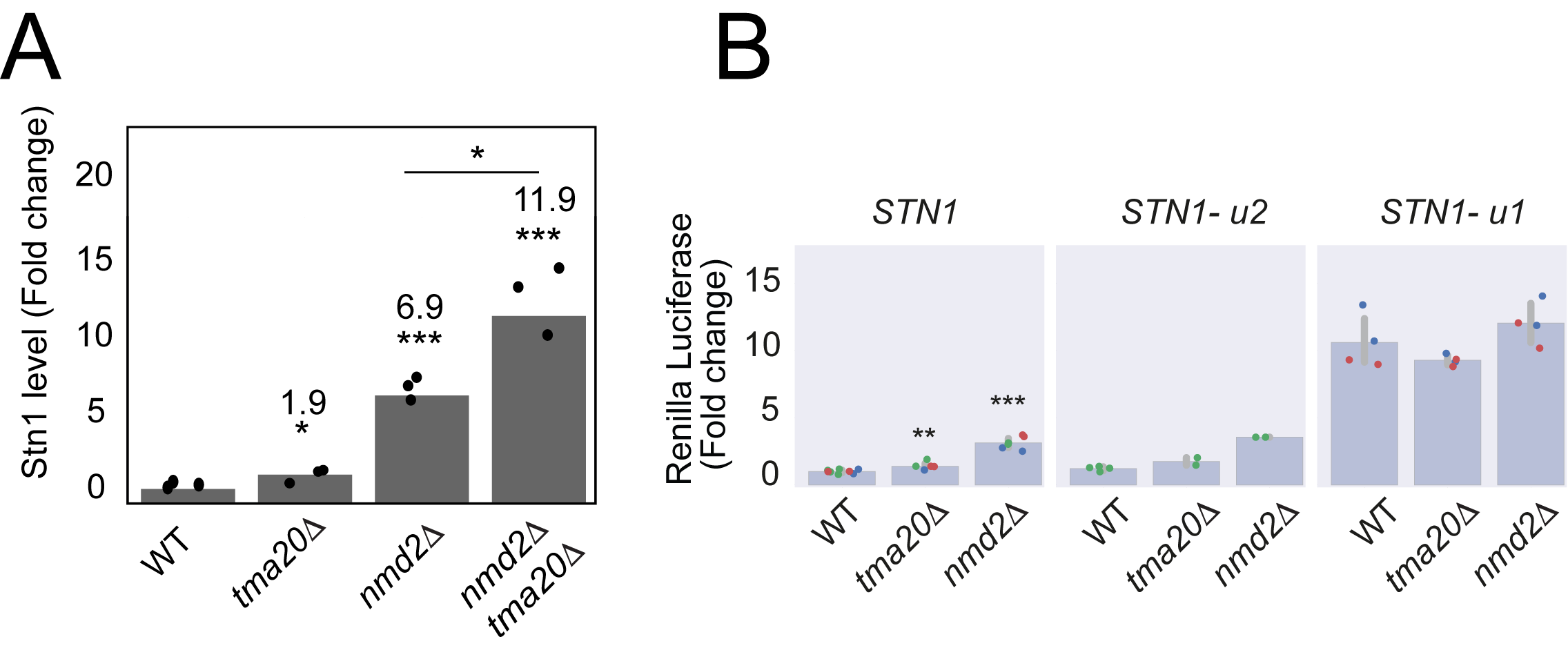

Supplement: S3 Fig — A) Quantification of Fig 3B and S9 Fig. B) Data in Fig 3D but normalized relative to expression of STN1 in WT cells. The points on the graph represent independent measurements, and are coloured according to the date that they were obtained. P values were calculated using an unpaired t-test (**) P < 0.01, (***) P < 0.001. (TIF) [file pgen.1007523.s003.tif]

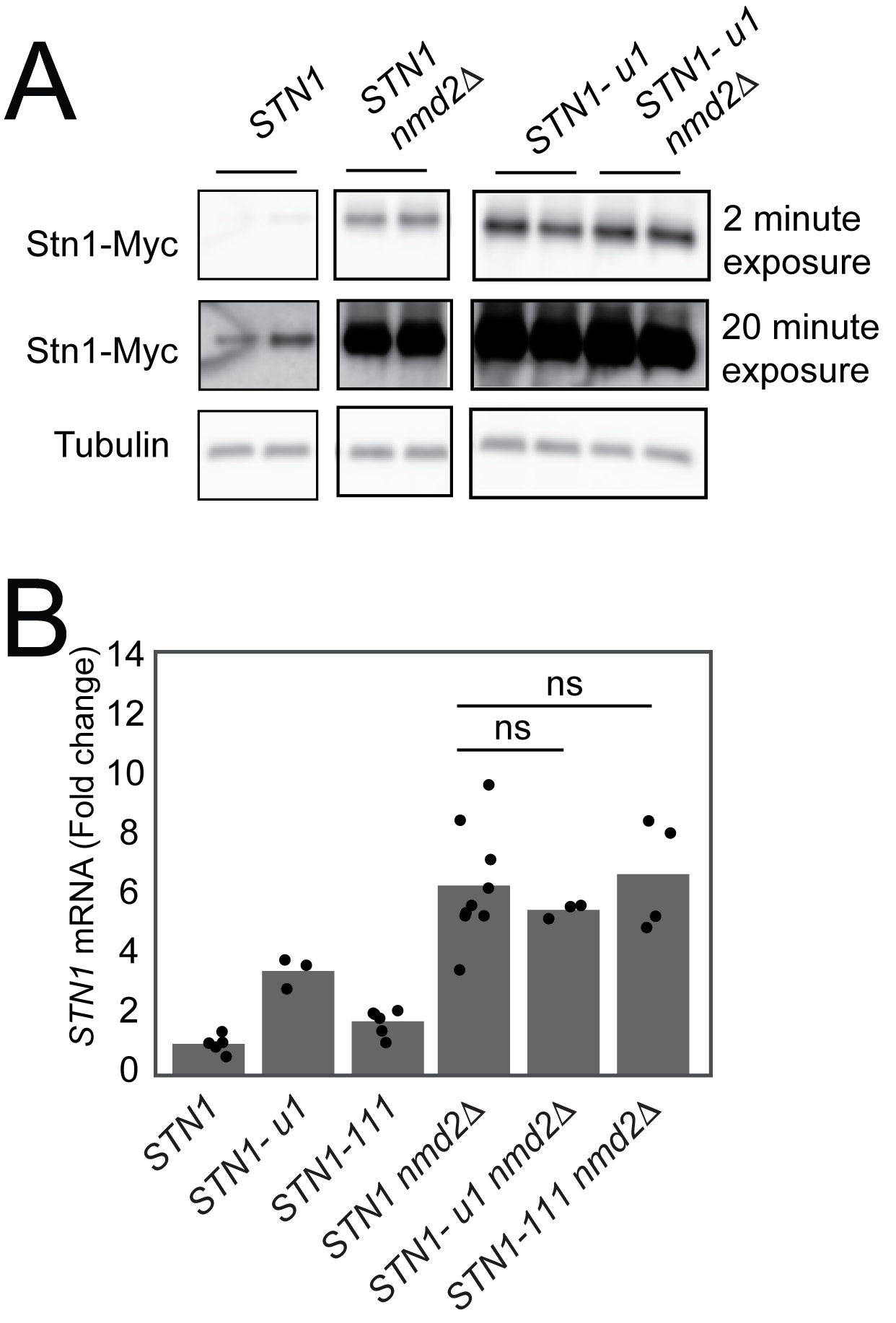

Supplement: S4 Fig — A) Western blot analysis of Stn1-Myc and Tubulin levels. The image is cropped from a single membrane. B) STN1 transcript levels compared with data also shown in Figs 1E, 3C and 4D. (TIF) [file pgen.1007523.s004.tif]

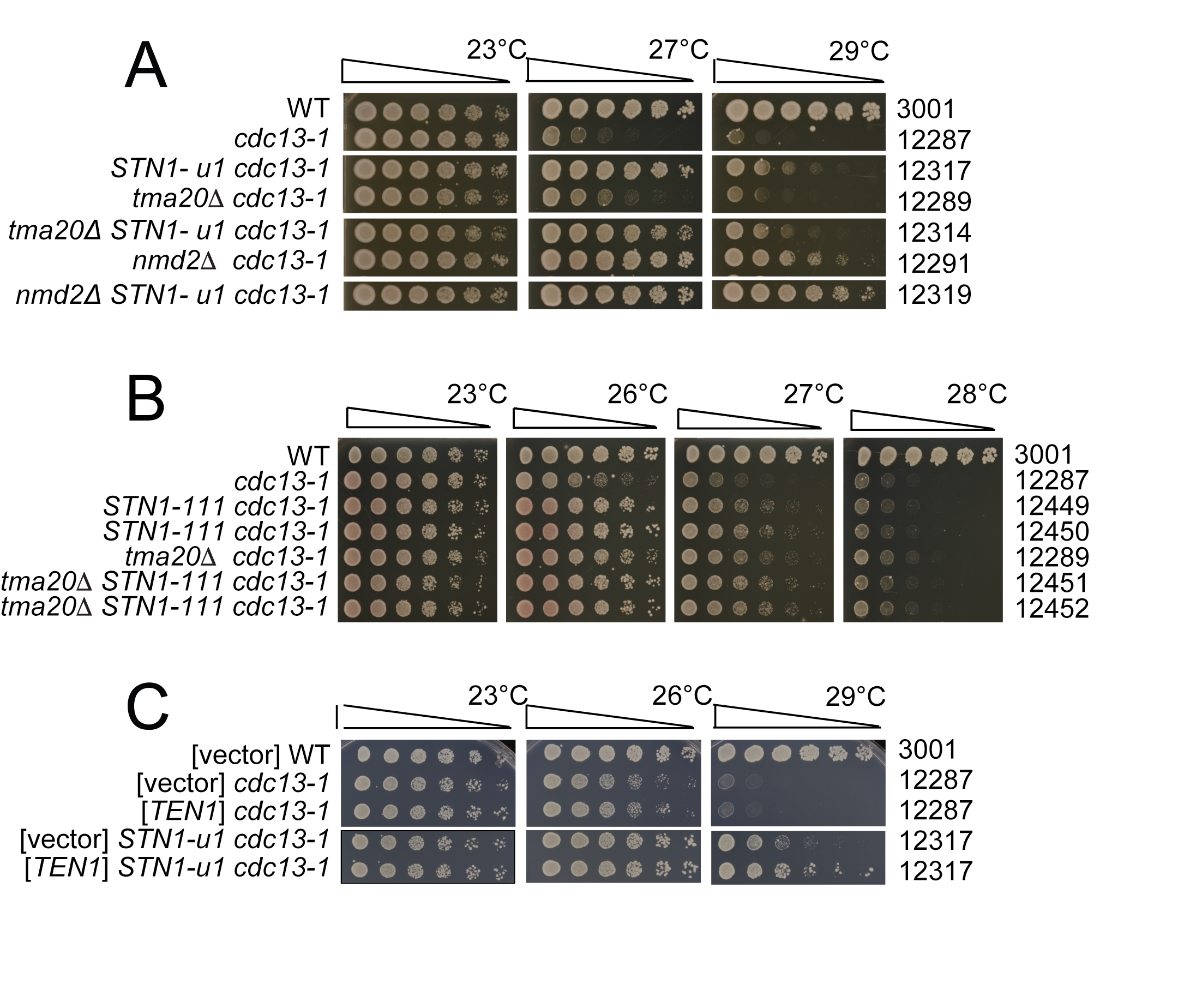

Supplement: S5 Fig — A-C) Saturated cultures of indicated genotypes were serially diluted, 5 fold, and spotted onto YEPD (A, B) or—LEU (C) solid media and incubated at indicated temperatures before being photographed. (TIF) [file pgen.1007523.s005.tif]

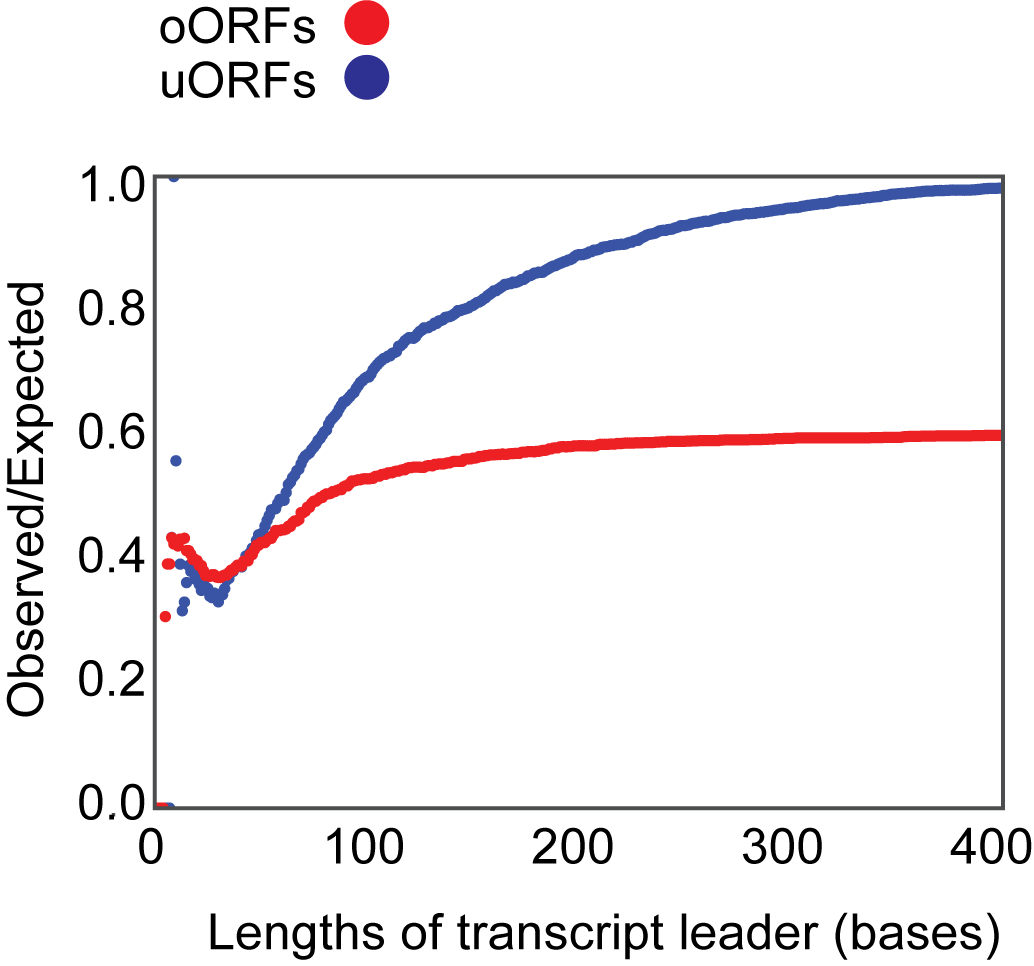

Supplement: S6 Fig — For each TL length the fraction of uORFs or oORFs observed in native sequences over those in randomized sequences was calculated (using the data from Fig 6B). (TIF) [file pgen.1007523.s006.tif]

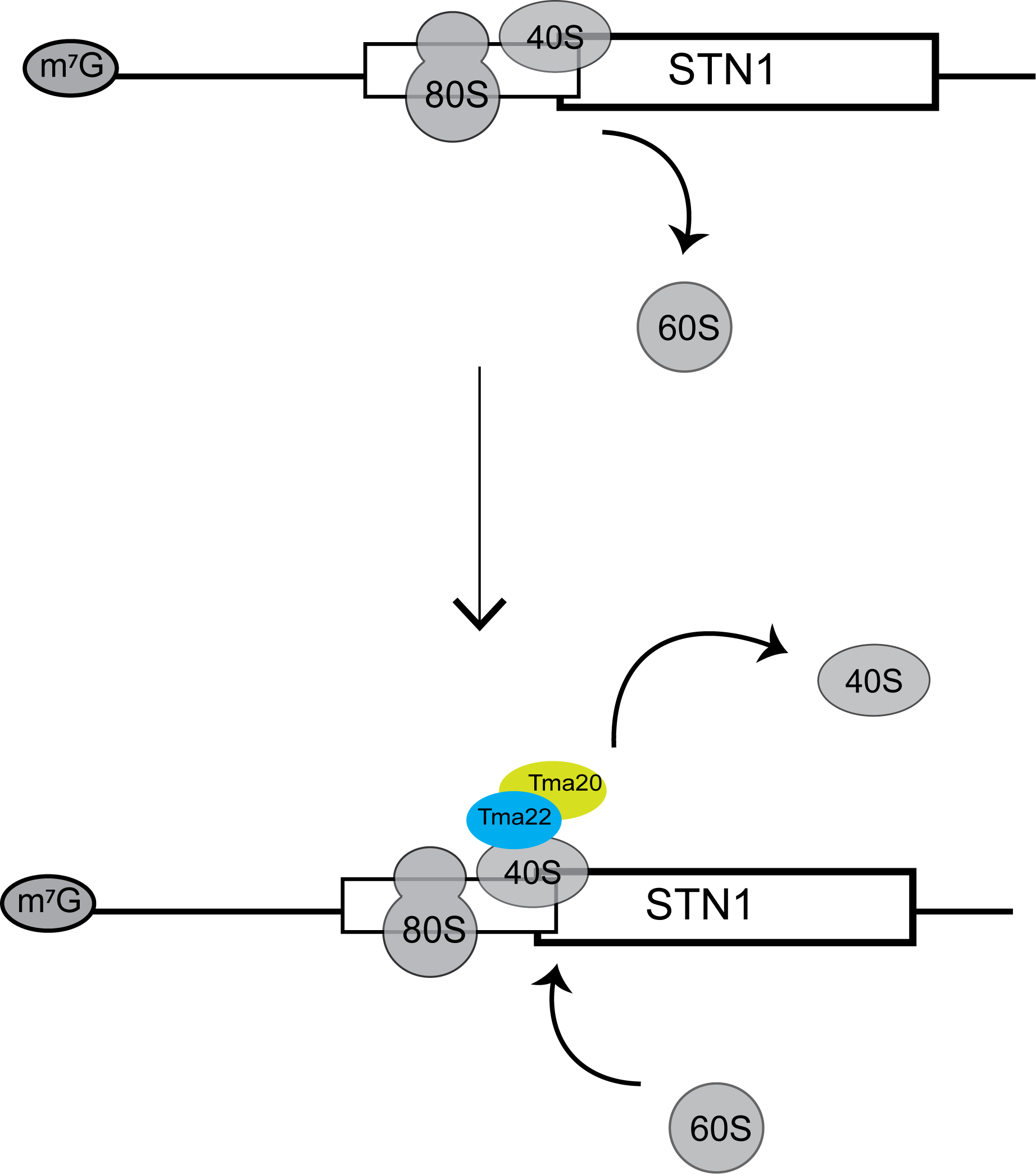

Supplement: S7 Fig — Following translation termination at the ribosome stop codon the 60S subunit dissociates from the termination complex. Tma20/Tma22 then promotes the dissociation of the 40S from the mRNA thus preventing re-initiation. The idea that Tma20/Tma22 reduce translation re-initiation is also supported by evidence showing that interaction of MCT-1Tma20/DENRTma22 with the 40S subunit is incompatible with the binding of the 60S ribosomal subunit to the 40S subunit [27]. (TIF) [file pgen.1007523.s007.tif]

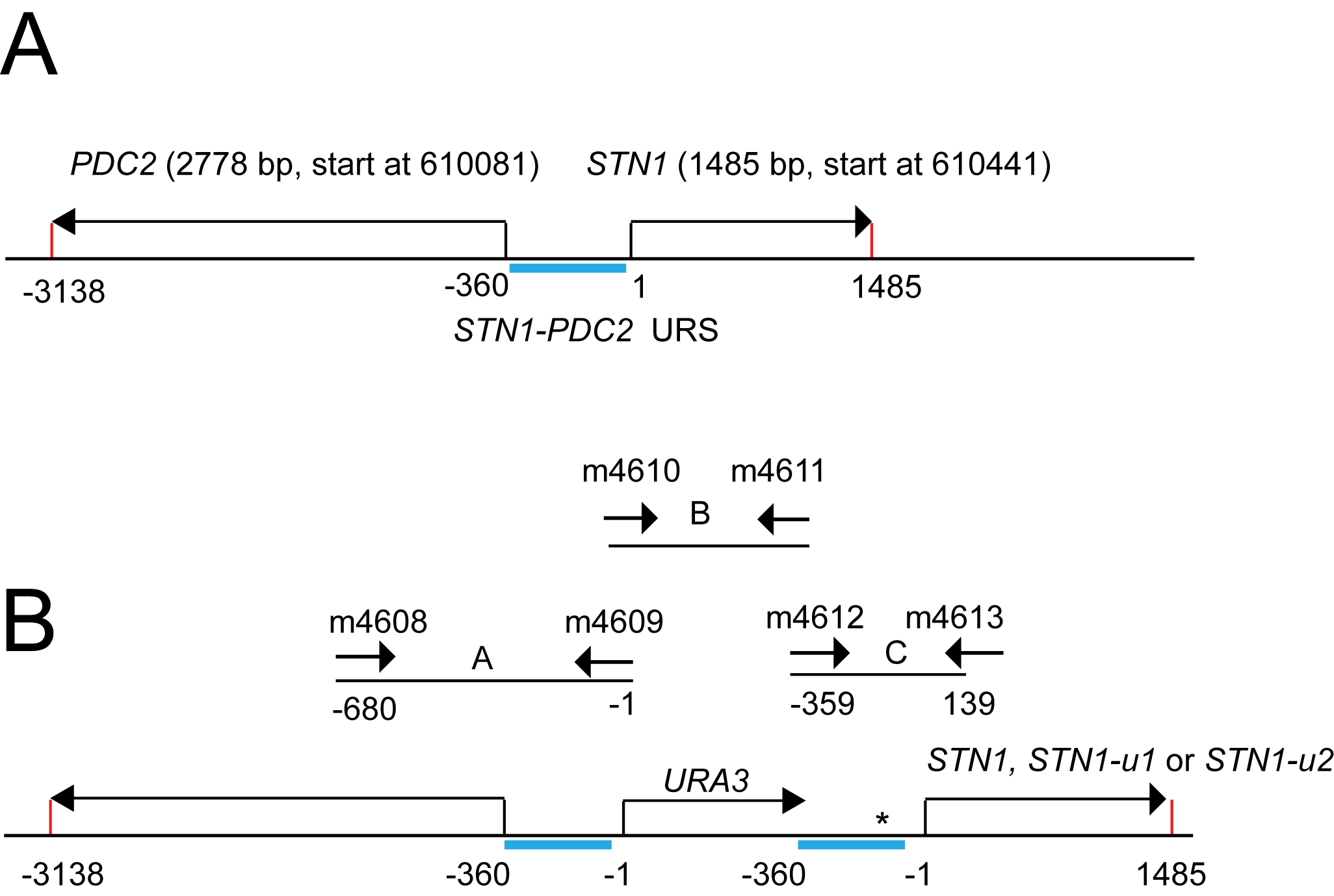

Supplement: S8 Fig — A) A map of the STN1-PDC2 locus. B) To ensure that expression of the adjacent gene (PDC2) was unaffected by mutations, the sequence separating STN1 and PDC2 was duplicated and separated by URA3. This was achieved by transforming yeast strains with an integrative plasmid assembled from three PCR products, labelled A, B and C. Fragment A was amplified using WT genomic DNA. Fragment B was amplified from pFA6URA3 (pDL1833). Fragment C was amplified from genomic DNA extracted from STN1 (STN1-u1 (DLY 11871) and STN1-u2 (DLY 11870) cells that contained additional unwanted point mutations in STN1 CDSs, using primers designed to separate STN1-u1 and STN1-u2 from the mutations in the CDSs. Primers used for PCR are indicated by mXXXX. (TIF) [file pgen.1007523.s008.tif]

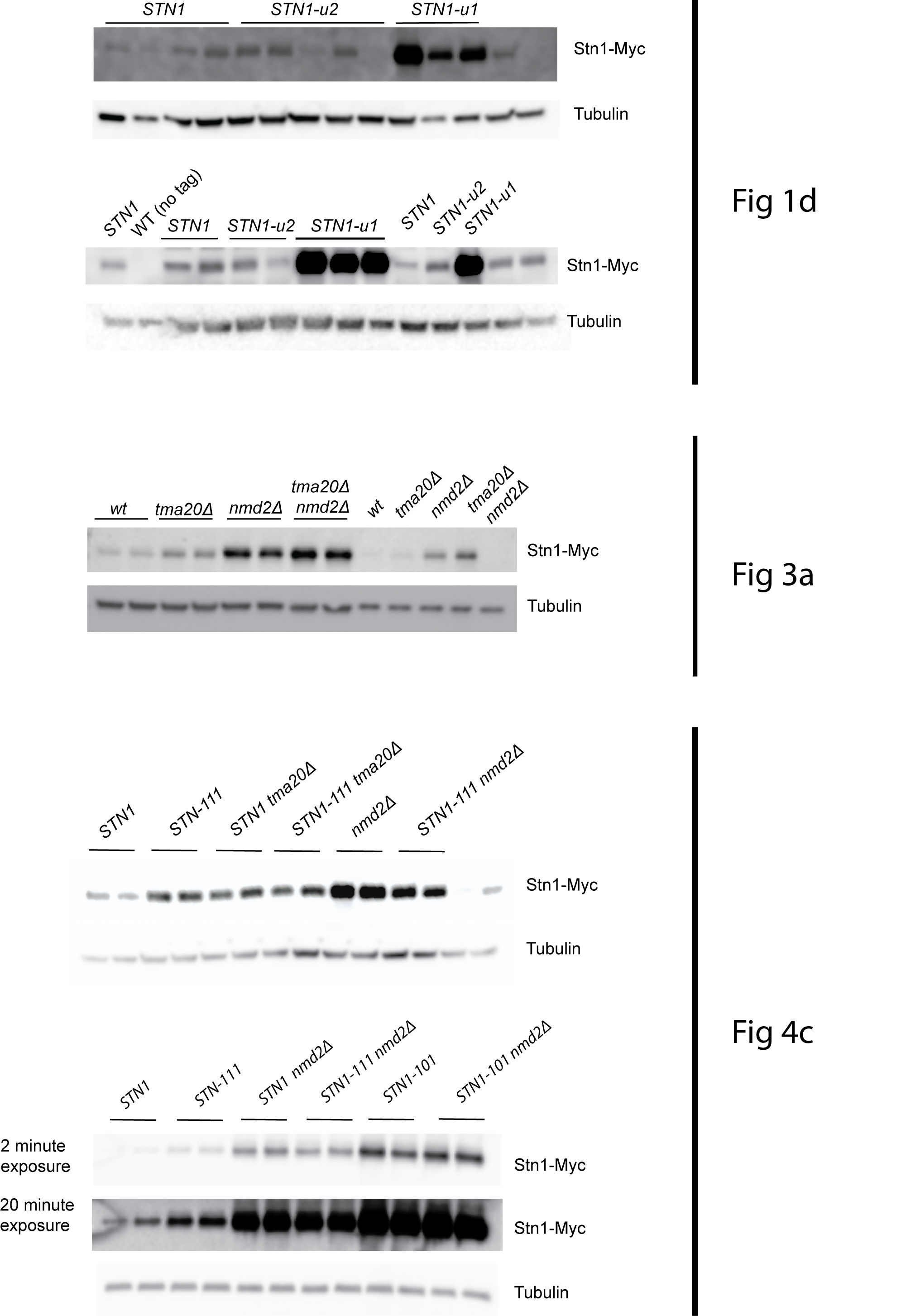

Supplement: S9 Fig — Blots providing additional data shown in Figs 1D, 4B and S3 Fig. (TIF) [file pgen.1007523.s009.tif]
