# Supplementary material for: Overlapping open reading frames strongly reduce human and yeast STN1 gene expression and affect telomere function
Source: PLoS Genet. 2018 Aug 1;14(8):e1007523. doi: 10.1371/journal.pgen.1007523 (PMC6089452; doi:10.1371/journal.pgen.1007523)
Supplement: S1 Table — (DOCX) [file pgen.1007523.s010.docx]

Table 1 – Table of Strains.

| **Strain**  **DLY** | **Genotype** | **Figure** | **Comments** |
| --- | --- | --- | --- |
| 3001 | *MATalpha ade2-1 trp1-1 can1-100 leu2-3,112 his3-11,15 ura3 GAL+ psi+ ssd1-d2 RAD5* | most | WT W303 |
| 8460 | *MAT a ade2-1 trp1-1 can1-100 leu2-3,112 his3-11,15 ura3 GAL+ psi+ ssd1-d2 RAD5* | most | WT W303 |
| 11813 | *MATa STN1-MYCx13-TRP1* | 1C, 1D, 1E, 3B, 4C | 5761 x 8624 |
| 11814 | *MATa STN1-MYCx13-TRP1* | 1C, 1D, 1E, 3B, 4C | 5761 x 8624 |
| 12400 | *MATa URA3-STN1-MYCx13-TRP1* | 1C, 1D, 1E, 4B-C | 11294 transformed with MYCx13 from 11813 |
| 12401 | *MATa URA3-STN1-MYCx13-TRP1* | 1C, 1D, 1E, 4B-C | 11294 transformed with MYCx13 from 11813 |
| 12402 | *MATa URA3-STN1-u2-MYCx13-TRP1* | 1C, 1D, 1E | 12309 transformed with MYCx13 from 11813 |
| 12403 | *MATa URA3-STN1-u2-MYCx13-TRP1* | 1C, 1D, 1E | 12309 transformed with MYCx13 from 11813 |
| 12404 | *MATa URA3-STN1-u1-MYCx13-TRP1* | 1C, 1D, 1E, 2C | 12325 transformed with MYCx13 from 11813 |
| 12405 | *MATa URA3-STN1-u1-MYCx13-TRP1* | 1C, 1D, 1E | 12325 transformed with MYCx13 from 11813 |
| 12352 | *MATa URA3-STN1* | 1E, 2C, S4B, 3C | 12294 x 5007 |
| 12414 | *MATa URA3-STN1-MYCx13-TRP1* | 1D, 4C | 12400 x 8624 |
| 12296 | *MATa URA3-STN1 tma20::KANMX nmd2::HIS3* |  | 12261 x 8529 |
| 12418 | *MATa URA3-STN1-MYCx13-TRP1 nmd2::HIS3* |  | 12400 x 8624 |
| 12295 | *MATa URA3-STN1 tma20::KANMX* | S4B | 12261 x 8529 |
| 12416 | *MATa URA3-STN1-MYCx13-TRP1 tma20::KANMX* |  | 12400 x 8624 |
| 12366 | *MATa URA3-STN1-u2* | 1E | 12309 x 5007 |
| 12416 | *MATa URA3-STN1-MYCx13-TRP1 tma20::KANMX* |  | 12400 x 8624 |
| 12374 | *MATa URA3-STN1-u1* | 1E, 2C, S4B | 12325 x 5007 |
| 12431 | *MATalpha URA3-STN1-u1-MYCx13-TRP1* |  | 12404 x 8624 |
| 1108 | *MATa cdc13-1* | 2A, 3A | Lab collection. |
| 12287 | *MATalpha STN1-URA3-cdc13-1* | 2A, S5A-C | 12261 x 8529 |
| 12317 | *MATa URA3-STN1-u1 cdc13-1* | 2A, S5A-C | 12263 x 8529 |
| 12313 | *MATalpha URA3-STN1-u1 cdc13-1* | 2A | 12263 x 8529 |
| 1412 | *MATa yku70::HIS3* | 2B | 12294 x 5007 |
| 12358 | *MATa URA3-STN1 yku70::LEU2* | 2B, 2C |  |
| 12359 | *MATalpha URA3-STN1 yku70::LEU2* | 2B, 2C | 12294 x 5007 |
| 12376 | *MATa URA3-STN1-u1 yku70::LEU2* | 2B, 2C | 12325 x 5007 |
| 12377 | *MATalpha URA3-STN1-u1 yku70::LEU2* | 2B, 2C | 12325 x 5007 |
| 12353 | *MATalpha URA3-STN1* | 2C | 12325 x 5007 |
| 12375 | *MATalpha URA3-STN1-u1* | 2C, S4B | 12325 x 5007 |
| 12361 | *MATa URA3-STN1 nmd2::HIS3* | 2C, S4B | 12294 x 5007 |
| 12360 | *MATalpha URA3-STN1 nmd2::HIS3* | 2C | 12294 x 5007 |
| 12378 | *MATa URA3-STN1-u1 nmd2::HIS3* | 2C, S4B | 12325 x 5007 |
| 12379 | *MATalpha URA3-STN1-u1 nmd2::HIS3* | 2C, S4B | 12325 x 5007 |
| 7026 | *MATa cdc13-1 tma20::KANMX* | 3A, S2C | Lab collection |
| 8538 | *MATalpha cdc13-1 tma22::KANMX* | 3A, S2C | Sporulation of DDY596 |
| 5106 | *MATa cdc13-1 nmd2::HIS3* | 3A | Lab collection |
| 8619 | *MATalpha cdc13-1 nmd2::HIS3 tma20::KANMX* | 3A | 6866 x 8518 |
| 8670 | *Mat a cdc13-1 nmd2::HIS3 tma22::KANMX* | 3A | 5256 x 8543 |
| 11815 | *MAT alpha STN1-MYCx13* | 3B | 5761 x 8624 |
| 11827 | *MAT alpha STN1-MYCx13 tma20::KANMX* | 3B | 5761 x 8624 |
| 11828 | *MAT alpha STN1-MYCx13 tma20::KANMX* | 3B, | 5761 x 8624 |
| 11819 | *MAT alpha STN1-MYCx13 nmd2::HIS3* | 3B | 5761 x 8624 |
| 11825 | *MAT alpha STN1-MYCx13 nmd2::HIS3 tma20::KANMX* | 3B | 5761 x 8624 |
| 11826 | *MAT alpha STN1-MYCx13 nmd2::HIS3 tma20::KANMX* | 3B | 5761 x 8624 |
| 4766 | *MATalpha nmd2::HIS3* | 3C, 3D |  |
| 8624 | *MATalpha nmd2::HIS3 tma20::KANMX* | 3C | 6866 x 8518 |
| 4528 | *MAT a nmd2::HIS3* | 3C, 3D | Lab collection |
| 8529 | *MATalpha tma20::KANMX* | 3C, 3D | 640 x 7023 |
| 8623 | *MATa nmd2::HIS3 tma20::KANMX* | 3C | 6866 x 8518 |
| 12322 | *MATalpha URA3-STN1-u1 tma20::KANMX* | S4B | 12263 x 8529 |
| 12356 | *MATalpha nmd2::HIS3* | S4B, 3C, 3D | 12294 x 5007 |
| 12326 | *MATa URA3-STN1-u1 tma20::KANMX* | S4B | 12263 x 8529 |
| 12325 | *MATa URA3-STN1-u1* | S4B | 12263 x 8529 |
| 12458 | *MATa URA3-STN1-111* | S4B, 3D | 12446 x 8620 |
| 12457 | *MATalpha URA3-STN1-111* | S4B, 3D | 12446 x 8620 |
| 12461 | *MATalpha URA3-STN1-111 nmd2::HIS3* | S4B | 12446 x 8620 |
| 12632 | *MATa URA3-STN1-111 nmd2::HIS3* | S4B | 12446 x 8620 |
| 8686 | *Mat alpha tma20::HPHMX* | 3C, 3D | 8567 x 7021 |
| 12468 | *MATa URA3-STN1-111 -MYCx13-TRP1* | 4B-C | 12444 x 8624 |
| 12469 | *MATalpha URA3-STN1-111-MYCx13-TRP1* | 4B-C | 12444 x 8624 |
| 12470 | *MATa URA3-STN1-111-MYCx13-TRP1 tma20::KANMX* | 4B-C | 12444 x 8624 |
| 12471 | *MATa URA3-STN1-111-MYCx13-TRP1 tma20::KANMX* | 4B-C | 12444 x 8624 |
| 11822 | *MAT alpha STN1-MYCx13 nmd2::HIS3* | 4B-C | 5761 x 8624 |
| 12475 | *MATalpha URA3-STN1-111-MYCx13-TRP1 nmd2::HIS3* | 4B-C | 12444 x 8624 |
| 12476 | *MATa URA3-STN1-111 -MYCx13-TRP1 nmd2::HIS3* | 4B-C | 12444 x 8624 |
| 12430 | *MATa URA3-STN1-u1-MYCx13-TRP1* | S4A | 12404 x 8624 |
| 12431 | *MATalpha URA3-STN1-u1-MYCx13-TRP1* | S4A | 12404 x 8624 |
| 12434 | *MATa URA3-STN1-u1-MYCx13-TRP1 nmd2::HIS3* | S4A | 12404 x 8624 |
| 12435 | *MATalpha URA3-STN1-u1-MYCx13-TRP1 nmd2::HIS3* | S4A | 12404 x 8624 |
| 12314 | *MATalpha cdc13-1 URA3-STN1-u1 tma20::KANMX* | S5A | 12263 x 8529 |
| 12289 | *MATalpha cdc13-1 URA3-STN1 tma20::KANMX* | S5A+B | 12261 x 8529 |
| 12319 | *MATa cdc13-1 URA3-STN1-u1 nmd2::HIS3* | S5A | 12263 x 8529 |
| 12449 | *MATalpha cdc13-1 URA3-STN1-111* | S5B | 12446 x 8620 |
| 12450 | *MATa cdc13-1 URA3-STN1-111* | S5B | 12446 x 8620 |
| 12451 | *MATa cdc13-1 URA3-STN1-111 tma20::KANMX* | S5B | 12446 x 8620 |
| 12452 | *MATalpha cdc13-1 URA3-STN1-111 tma20::KANMX* | S5B | 12446 x 8620 |
| 11369 | *cdc13-1 tma20::KANMX* | S2C | 11284 x 8716 |
| 8539 | *MATa cdc13-1 tma22::KANMX* | S2C, 2A | Sporulation of DDY596 |
| 8637 | *MATalpha cdc13-1-int tma22::HPH tma20::KANMX* | S2C | 8566 x 8529 |
| 8638 | *MATa cdc13-1-int tma22::HPH tma20::KANMX* | S2C | 8566 x 8529 |
| 12291 | *MATalpha URA3-STN1 cdc13-1 nmd2::HIS3* | S5A | 12261 x 8529 |

Footnote:  *STN1-u1, STN1-u2* and *STN1-111* increases protein and mRNA levels. *STN1-u1* is a dominant allele (Fig S1), and on this basis we infer that *STN1-u2* and *STN1-111* are also dominant.
