# Supplementary material for: Overlapping open reading frames strongly reduce human and yeast STN1 gene expression and affect telomere function
Source: PLoS Genet. 2018 Aug 1;14(8):e1007523. doi: 10.1371/journal.pgen.1007523 (PMC6089452; doi:10.1371/journal.pgen.1007523)
Supplement: S2 Table — (DOCX) [file pgen.1007523.s011.docx]

| **Primers for Gene Deletion** | | |  |
| --- | --- | --- | --- |
|  |  |  |  |
| m2858 | TTCAAGAGCTAAACTAAAGAAAAGCATATTGCATAAAATGCGGATCCCCGGGTTAATTAA | *TMA22* |  |
| m2862 | TCTCCCAGAACGGTGCTATTACATATTTATGGATTGCTTAGAATTCGAGCTCGTTTAAAC | *TMA22* |  |
|  |  |  |  |
|  |  |  |  |
| **Primers for creating plasmids** | |  |  |
|  |  |  |  |
| **Primers** | **Sequence** | **Target** | **Template DNA** |
| m3926 | GCGCCGGGCCTTTCTTTATGTTTTTGGCGTCTTCCATTATATTTGTTGTAAAAAGTAGATAATTAC | *PGK1* promoter | 3001 DNA |
| m3812 | ACGGCCAGTGAGCGCGCGTAATACGACTCACTATAGGGCGGTCACGACGTTGTAAAACGAC | *PGK1* promoter | 3001 DNA |
| m3829 | GAGAAACTTTTTTAATTCGTAAGCTTGATATCGAATTCCTGCAGGGTAAAATGGAAGACGCCAAAAAC | *Firefly Luciferase* | pDL1897 |
| m3830 | GCTGGAGCTCCACCGCGGTGGCGGCCGCTCTAGAACTAGTTTTACAATTTGGACTTTCCGCC | *Firefly Luciferase* | pDL1897 |
| m3962 | CCTCATAAAGGCCAAGAAGGGCGGAAAGTCCAAATTGTAAGCTTTTCCTTTGTCGATATCATGTAATTAG | *CYC1* terminator | 3001 DNA |
| m3963 | AGCTTGCAAATTAAAGCCTTC | *CYC1* terminator | 3001 DNA |
| m3964 | GAGAAGGTTTTGGGACGCTCGAAGGCTTTAATTTGCAAGCTGGTGTGGTCAATAAGAGCGACC | *ADH1* terminator | 3001 DNA |
| m3965 | GCGAATTTCTTATGATTTATGATTTTTATT | *ADH1* terminator | 3001 DNA |
| m3966 | TTATAACTTATTTAATAATAAAAATCATAAATCATAAGAAATTCGCTTATGTTTTTGGCGTCTTCGAGC | *Renilla luciferase* | pDL1659 |
| m3967 | GGGAACAAAAGCTGGAGCTCCACCGCGGTGGCGGCCGCTCTAGATGACTTCGAAAGTTTATGATCCAG | *Renilla luciferase* | pDL1659 |
| m4608 | ATATCAGTTATTACCCGGGCTGTTTAAACGAGCTCGAATTCGCTGGTTATAATAAAGCTGTCTC | *STN1/PDC2* URS and first 139 bps of *STN1* | genomic DNA |
| m4609 | CAAGCTCGGAATTAACCCTCACTAAAGGGAACAAAAGCTGCTCTCATCCGGTACTTTAAATC | *STN1/PDC2* URS and first 139 bps of *STN1* | genomic DNA |
| m4610 | AAAGAGCAATAAGCTGGCTTTTAATAATGGGTTGGAGCCGGATCCCCGGGTTAATTAAG | *URA3* in including endogenous URS and terminator in the same orientations *STN1* | pDL1833 |
| m4611 | TAACACCAAGCAGTAAAGAGACAGCTTTATTATAACCAGCGAATTCGAGCTCGTTTAAAC | *URA3* in including endogenous URS and terminator in the same orientationas *STN1* | pDL1833 |
| m4612 | GGCCAGTGAATTGTAATACGACTCACTATAGGGCGAATTGGTGCCAAACAGCTTGTGCAG | *STN1/PDC2* URS | 3001 DNA |
| m4613 | GCTTTATGGAGGATCTGGCGCGCCTTAATTAACCCGGGGATCCGGCTCCAACCCATTATTAAAAG | *STN1/PDC2* URS | 3001 DNA |
| m4563 | AACGTCAAAGGGCGAAAAACCGTCTATCAGGGCGATGGCCGGTATACTGGTAACACAAATAC | *TEN1* | 3001 DNA |
| m4564 | CCAAGCGCGCAATTAACCCTCACTAAAGGGAACAAAAGCTGATCTAACCCAGAATTATTGAGAATCAG | *TEN1* | 3001 DNA |
| m4671 | CTCTATCGATAGGTACCGAGCTCTTACGCGTGCTAGCCCGGACCCAACCTCTAGGAGGCCCAGG | human STN1 URS | cDNA |
| m4672 | GGACACAGAAATGAGGATGCAATGCG | human STN1 URS | cDNA |
|  |  |  |  |
|  |  |  |  |
|  |  |  |  |
| **Primers for qPCR** | | |  |
|  |  |  |  |
| m1172 | CAGACCGAACTCGGTGATTT | *BUD6* |  |
| m1173 | TTTTAGCGGGCTGAGACCTA | *BUD6* |  |
| m1734 | TCGAGCAACTGCAAGAAGAA | *STN1* |  |
| m1735 | CGAAATGACAAGGAATGCAC | *STN1* |  |
