# Supplementary material for: Overlapping open reading frames strongly reduce human and yeast STN1 gene expression and affect telomere function
Source: PLoS Genet. 2018 Aug 1;14(8):e1007523. doi: 10.1371/journal.pgen.1007523 (PMC6089452; doi:10.1371/journal.pgen.1007523)
Supplement: S3 Table — (DOCX) [file pgen.1007523.s012.docx]

Table 3 – List of plasmids

| 1659 | plasmid containing Renilla Luciferase | From Jeremy Brown |  |
| --- | --- | --- | --- |
| 987 | Southern Blot probe | (1) |  |
| 1897 | pGL3 basic Firefly luciferase expression vector | from Neil Perkins |  |
| 1896 | pRL-TK Renilla luciferase expression vector | from Neil Perkins |  |
| 1833 | pFA6-URA3 | from Jon Houseley |  |
|  |  |  |  |
|  |  |  |  |
| 1701 | pRS415 with PGK1 promoting Firefly | Frag1 - PGK1 promoter | m3926 and m3812 from DLY 3001 |
|  |  | Frag 2 - Firefly ORF | m3829 and m3830 pDL1897 |
| 1728 | PGK1 drives Firefly luciferase YFG drives Renilla luciferase | CYC1 terminator sequence | m3962 and m3963 from DLY 3001 DNA |
|  |  | ADH1 terminator | m3964 and m3965 from DLY 3001 DNA |
|  |  | Renilla luciferase | m3966 and m3967 from pDL1659 |
|  |  | backbone - pDL1701 digested with Spe1 and BamH1 |  |
|  |  |  |  |
|  |  |  |  |
| 1850 | STN1 Reporter | STN1 promoter region | m4449 and m4450 from DLY 3001 DNA |
|  |  | backbone - pDL1728 digested with Not1 |  |
| 1851 | Reporter (STN1-u2) (aka STN1-M1V, 102) | Frag 1 - STN1 M1V Reporter (STN1-u2) | m4035 and m4451 used to amplify part1 from pDL1850 |
|  |  | Frag 2 - STN1 M1V Reporter (STN1-u2) | m4038 and m4452 used to amplify part 2 from pDL1850 |
|  |  | backbone - pDL1728 digested with Not1 |  |
| 1852 | Reporter (STN1-u1) aka STN1-M2V, 101) | Frag 1 - STN1 M2V Reporter (STN1-u1) | m4035 and m4465 used to amplify part1 from pDL1850 |
|  |  | Frag 2 - STN1 M2V Reporter (STN1-u1) | m4038 and m4466 used to amplify part 2 from pDL1850 |
|  |  | backbone - pDL1728 digested with Not1 |  |
|  |  |  |  |
|  |  |  |  |
|  |  |  |  |
| 1867 | Integrate STN1 with URA3 in the URS into genome | Frag 1 - STN1 URS and first 139 bps of STN1 | m4608 + m4609 from DLY 3001 |
|  |  | Frag 2 - URA3 in including endogenous URS and terminator in the same orientations STN1 | m4610 + m4611 from pDL1833 |
|  |  | Frag 3 - PDC2 URS | m4612 + m4613 from DLY 3001 |
|  |  | pDL 452 digested with BamH1 and Xho1 |  |
| 1868 | Integrate STN1-u2 with URA3 in the URS into genome | Frag 1 - STN1 URS and first 139 bps of STN1 | m4608 + m4609 from 11870 DNA |
|  |  | Frag 2 - URA3 in including endogenous URS and terminator in the same orientations STN1 | m4610 + m4611 from pDL1833 |
|  |  | Frag 3 - PDC2 URS | m4612 + m4613 from 3001 DNA |
|  |  | backbone - pDL452 (cut with BamH1 and Xho1) |  |
| 1869 | Integrate STN1-u1 with URA3 in the URS into genome | Frag 1 - STN1 URS and first 139 bps of STN1 | m4608 + m4609 from 11871 DNA |
|  |  | Frag 2 - URA3, including endogenous URS and terminator | m4610 + m4611 from pDL1833 |
|  |  | Frag 3 - PDC2 URS | m4612 + m4613 from 3001 DNA |
|  |  | backbone - pDL452 (cut with BamH1 and Xho1) |  |
|  |  |  |  |
| 1688 | TEN1 | TEN1 (800bps before start and 400bps after stop of CDS) | m4563 and m4564 from 3001 purified DNA |
|  |  | pDL16 digested at MCS |  |
|  |  |  |  |
|  |  |  |  |
| 1898 | Human STN1-Fluc | Fragment 1 - (fragment of STN1) | amplified using m4671 and m4672 from cDNA |
|  |  | Fragment 2- was ordered from eurofins as dsDNA GCCACTTTGACCCACGTTAAACGCATTGCATCCTCATTTCTGTGTCCCATCTAGATGCTTGACTCAGTGATGCAGAACCTTTCAGAGTTAGCTGGAAGCCACAGCCCTGCCTCTTGATGGAAGACGCCAAAAACATAAAGAAAGGCCCGGCGCCATTC |  |
|  |  | Fragment 3- is pGL3 1897 (Linearized this with Xho1 and HindIII) |  |
| 1898 | Human STN1-no-oORF-Fluc | Fragment 1 - (fragment of STN1) | amplified using m4671 and m4672 from cDNA |
|  |  | Fragment 2 was ordered from eurofins as dsDNA GCCACTTTGACCCACGTTAAACGCATTGCATCCTCATTTCTGTGTCCCATCTAGATACTTGACTCAGTGATACAGAACCTTTCAGAGTTAGCTGGAAGCCACAGCCCTGCCTCTTGATGGAAGACGCCAAAAACATAAAGAAAGGCCCGGCGCCATTC |  |
|  |  | Fragment 3- is pGL3 1897 (Linearized this with Xho1 and HindIII) |  |

1. Tsubouchi H, Ogawa H. Exo1 roles for repair of DNA double-strand breaks and meiotic crossing over in Saccharomyces cerevisiae. Mol Biol Cell. 2000;11(7):2221-33.
